# Abstracts from “Chronic viral infection and cancer, openings for vaccines” virtual symposium of the TechVac network

**DOI:** 10.1186/s13027-022-00435-1

**Published:** 2022-07-08

## I1. Symposium Opening. Chronic viral infections and cancer, openings for vaccines

### Maria G Isaguliants^1^, Maria Lina Tornesello^2^, and Franco M Buonaguro^2^

#### ^1^Riga Stradins University, Riga, Latvia; ^2^Molecular Biology and Viral Oncology Unit, Istituto Nazionale Tumori – IRCCS Fondazione Pascale, Napoli, Italy

##### Presenting author: Maria G Isaguliants (maria.issagouliantis@rsu.lv)

*Infectious Agents and Cancer* 2022, **17(Suppl 1)**: I1

Approximately one out of eight human cancers has viral etiology. Viral cancers present unique opportunities for prophylaxis, diagnosis, and therapy, as demonstrated by the success of HBV and HPV vaccines and HCV antivirals in decreasing the incidence of tumors that are caused by these viruses. For other chronic viral infections and related cancers, problems still dominate over the progress. Prophylactic HPV vaccination does not cure already established infections, urgently requesting development of therapeutic HPV vaccines. Several are in pipeline, but still far from actual application for treatment of HPV-related cancers. Development of HCV vaccines is in progress, but is hampered by HCV variability and absence of longitudinal sterilizing anti-viral response. The success of antiretroviral therapy in controlling HIV-1 replication and immune reconstitution did not lead to significant reduction of HIV-1 associated cancers indicating alternative mechanisms of their development, not connected to immune suppression.

Symposium aims were to unravel the progress in the studies of the molecular pathogenesis of chronic viral infections, and summarize common mechanisms of action of human oncogenic viruses, such as the activity of viral oncoproteins, induction of genomic instability, support of chronic inflammation, and modulation of tumor microenvironment. Topics that received special attention were: (i) virus-induced alterations in the functions of immune system, (ii) induction of innate and adaptive immune responses, (iii) the relationship between the immune response to viral infections; and (iv) metabolism from the infected and cancerous cells to the whole body level. In-depth understanding of these processes creates innovative openings to the development of novel viral vaccines and immunotherapies.

## Session “molecular pathogenesis of chronic viral infections”

### PL1. Chronic viral infections and oxidative stress

#### Alexander V. Ivanov

##### Engelhardt Institute of Molecular Biology, Russian Academy of Sciences, Moscow, Russia

##### Presenting author: Alexander V. Ivanov (aivanov@yandex.ru)

*Infectious Agents and Cancer* 2022, **17(Suppl 1)**: PL1

**Background: **Chronic viral infections are the etiological agents of a wide spectrum of diseases that often affect quality of life of a patient and even impose risk of mortality. This group of infections include human immunodeficiency virus (HIV), hepatitis B, C, and delta viruses (HBV, HCV, HDV), papilloma virus (HPV), various herpes viruses and others. HIV infects lymphocytes and affect immune system of a patient thus inducing acquired immunodeficiency syndrome (AIDS), hepatitis viruses trigger liver inflammation and liver fibrosis that lead to development of cirrhosis and cancer – the end-stage liver diseases. HPV is known to induce several types of tumors that also impose threat for a patient. However, mechanisms of pathogenesis of these infections are not fully understood. The aim is to review the field of redox biology of viral infections in the context of the abovementioned viral infections in order to unveil importance of reactive oxygen species for replication of the viruses and for development of virus-associated diseases.

**Topic overviewed: **Various acute and chronic viral infections are accompanied by enhanced production of reactive oxygen species (ROS). They include respiratory viruses, hepatitis B and C viruses (HBV, HCV), human immunodeficiency virus (HIV), human papilloma virus (HPV), herpesviruses and others. Levels of oxidative stress markers correlate with virus-associated pathologies: inflammation, fibrosis, and tumor incidence. Moreover, ROS play important role in activation of various signaling pathways that promote production of proinflammatory cytokines, tumor invasiveness and angiogenesis, adaptation to hypoxic conditions etc. In recent years many endeavors are made to unveil sources of ROS that are activated by viruses and to reveal cellular and viral proteins that undergo redox-dependent post-translational modifications. A separate line of studies is to evaluate role of changes in redox status and ROS in replication of viruses and especially in their latency. Finally, there are multiple data showing that viruses interfere with cellular antioxidant defense pathways and that antioxidants both affect virus replication and suppress development of virus-associated pathologies.

**Conclusions: **Increased ROS production during various viral infections is one of the major factors for development of virus-associated inflammation, fibrosis and cancer. Understanding of the mechanisms by which viruses interfere with redox state of a host cell is critical for development of approaches for treatment of virus-associated diseases.

**Acknowledgements: **Russian science foundation (grant #19-14-00197).

### PL2. Metabolic signatures of chronic viral infections

#### Birke Bartosch

##### University of Lyon, Université Claude Bernard Lyon 1, INSERM 1052, CNRS 5286, Centre Léon Bérard, Centre de recherche en cancérologie de Lyon, Lyon, 69434, France

###### Presenting author: Birke Bartosch (birke.bartosch@inserm.fr)

*Infectious Agents and Cancer* 2022, **17(Suppl 1)**: PL2

A significant portion of cancer in the world is due to chronic viral infections. Viruses induce oncogenesis by targeting the same pathways known to be responsible for neoplasia in tumor cells, such as control of cell cycle progression, cell migration, proliferation and evasion from cell death and the host’s immune defense. In addition, metabolic reprogramming has been identified over a century ago as a requirement for growth of transformed cells. Renewed interest in this topic has emerged recently with the discovery that basically all metabolic changes in tumor cells are finely orchestrated by oncogenes and tumor suppressors. Indeed, cancer cells activate biosynthetic pathways in order to provide them with sufficient levels of energy and building blocks to proliferate and with metabolites that allow cancer cells to modulate the micro-environment in their favor. Interestingly, viruses introduce into their host cells similar metabolic adaptations, and importantly, it seems that they depend on these changes for their persistence and amplification. The central carbon metabolism, for example, is not only frequently altered in tumor cells but also modulated by human papillomavirus, hepatitis B and C viruses, Epstein–Barr virus and Kaposi’s Sarcoma-associated virus. Moreover, adenoviruses (Ad) and human cytomegalovirus, which are not directly oncogenic but present oncomodulatory properties, also divert cellular metabolism in a tumor cell-like manner. Overall, metabolic reprogramming appears to be a hallmark of viral infection and provides an interesting therapeutic target, in particular, for oncogenic viruses.

### PL3. Chronic viral infections and DNA repair system of the cell

#### Marina B. Gottikh*, Semyon O. Galkin, Ekaterina A. Ilgova, Ekaterina A. Nefyodova, Andrey N. Anisenko

##### Lomonosov Moscow State University, Moscow, Russia

###### Presenting author: Marina B. Gottikh (gottikh@belozersky.msu.ru)

*Infectious Agents and Cancer* 2022, **17(Suppl 1)**: PL3

**Background: **To detect DNA damage sites, which are deleterious for genome integrity, and to coordinate the repair process, eukaryotes have evolved a complex signaling network called the DNA damage response (DDR), which is principally mediated by the PI3K kinases: ATM, ATR and DNA-PK, and by the poly (ADP-ribose) polymerases. Numerous viruses induce DNA damage and genetic instability in host cells during their lifecycles, and some species also manipulate components of the DDR. The aim of this review was to provide a brief description of the interconnection between human DDR and chronic viral infections. In addition, we present the results of our studies on the involvement of components of the NHEJ repair system in HIV replication and the development of a new type of HIV-1 inhibitors.

**Topics overviewed: **Viruses have developed various strategies to modulate DDR, and some of them use DDR for their own benefit. This is the most interesting case as it gives us some new approaches to suppressing viral infection. Polyomaviruses induce and exploit the ATM and ATR signaling pathways. Inhibition of DDR kinases reduced the DNA replication of Merkel cell polyomavirus and BK polyomavirus. DDR is also involved in the life cycle of hepatitis B virus (HBV), chronically infecting hundreds of millions of people. The reason for the chronicity is a special form of the virus genome—circular covalently closed DNA (cccDNA), remaining insensitive to antiviral therapy. Inhibition of ATR and its downstream signaling factor CHK1, but not of ATM, decreased cccDNA formation during de novo HBV infection, and intracellular cccDNA amplification. Hepatitis C virus NS3 protein is shown to interact with DNA repair factors, Werner syndrome protein and Ku70, which is a component of DNA-PK, thereby diminishing the repair efficiency of NHEJ initiated by DNA-PK.

HIV-1 can exploit the NHEJ pathway for its benefit. Integration of viral DNA into a host genome, performed by viral enzyme integrase, results in single-stranded gaps in the cell DNA that must be repaired. We have analyzed the involvement of ATM, ATR and DNA-PK in the post‐integrational gap repair (PIR), and found that inhibition of DNA-PK and ATM, but not of ATR, significantly reduces the PIR efficiency. This is a rather surprising result, given that both kinases are sensors for double-strand breaks that are not formed during integration. We have shown that DNA-PK is recruited to integration sites due to binding to Ku70, and the binding disruption disturbs HIV-1 replication. Structural investigation of the binding site of these proteins allowed us to carry out molecular docking and find inhibitors of the proteins’ interaction. The leader compound is capable of suppressing PIR at micromolar concentrations. Further optimization of the inhibitor structure is undoubtedly required, but nevertheless we can talk about a new approach to HIV suppression by blocking PIR.

**Conclusions: **Elucidation of the interactions between viruses and DDR is important both for understanding the modulation of host cell functions by these pathogens and for developing new approaches to combating viral infections. This work was supported by RSF grants 19‐74‐10021, 17-14-01107 and RFBR grant 18-29-08012.

### O1. Hepatitis delta virus antigens trigger ROS production and activate antioxidant response factors

#### Artemy Fedulov*, Olga Ivanova, Vladimir Valuev-Elliston, Olga Smirnova, Alexander V. Ivanov

##### Engelhardt Institute of Molecular Biology, Russian Academy of Sciences, Moscow, Russia

###### Presenting author: Artemy Fedulov (fedulovtt@gmail.com)

*Infectious Agents and Cancer* 2022, **17(Suppl 1)**: O1

**Background: **Hepatitis delta virus (HDV) is a satellite RNA virus that infects HBV-infected hepatocytes. Virion is composed of HBV surface proteins and contains a negative-sense ssRNA genome. The virus utilizes host cell RNA polymerases I and II for replication and transcription of the genome. The viral 0.8 kb mRNA encodes 196 amino acid protein (i.e.small delta antigen, S-HDAg) of 24 kDa. At late stages of virus life cycle cellular adenosine deaminase (ADAR-1) edits anticodon of HDV mRNA leading to extension of the open reading frame with additional 19 triplets. It results in 27 kDa protein referred to as large delta antigen (L-HDAg). S-HDAG acts as an activator in viral genome replication while L-HDAg is important for assembly and secretion of HDV virions. Despite more than 40 years of HDV studies, mechanisms of virus pathogenesis are still mostly unknown. For other hepatitis viruses it was shown that oxidative stress is one of the major factors of development of liver dysfunction. In 2013 Williams et al. demonstrated a significant increase in reactive oxygen species (ROS) production in cells overexpressing L-HDAg resulting in activation of two key redox-dependent proinflammatory transcription factors: NF-kB and STAT3. However, methodologically this paper is not complete, as a very few approaches were used. The same authors also claimed that HDV antigens did not trigger unfolded protein response (UPR) though a single experiment presented showed the opposite. The aim of this study was to re-evaluate the impact of HDV and its antigens on ROS production, expression of ROS-generating enzymes, status of antioxidant defense system and, lastly, on possible ER stress and UPR.

**Materials and methods: **Huh7.5 cells were transfected with plasmids ensuring overexpression of HDV antigens or setting autonomous replication of viral RNA. ROS production was evaluated using redox-sensitive fluorescent dyes 2’,7,-dichlorodihydrofluorescein diacetate (DCFH2DA), dihydroethidium (DHE) and MitoSOX that show redox status and production of superoxide anions in a cell or in mitochondria, respectively. Gene expression was monitored by RT-qPCR and Western blotting.

**Results: **We have shown that overexpression of both HDV antigens in Huh7.5 hepatoma cells as well as the replication of viral genome lead to altered redox status and enhanced production of superoxide anions both in cytoplasm and mitochondria. It was accompanied by increased transcription of NADPH oxidases 1 and 4, cytochrome P450 2E1 and ER oxidoreductin 1a (Ero1a) that generate both superoxide and hydrogen peroxide. Moreover, both antigens as well as HDV RNA replication led to activation of the Nrf2 transcription factor that controls expression of various antioxidant enzymes. The cells overexpressing these proteins were characterized by increased levels of NADPH oxidoreductase 1 (NQO1) and heme oxygenase 1 (HO-1). Finally, both HDV antigens triggered unfolded protein response, as revealed by increased expression of various UPR-dependent genes.

**Conclusions: **So, we showed that HDV antigens alone or in the context of replication of viral RNA trigger ROS production, activate antioxidant Nrf2/ARE pathway and induce unfolded protein response. Future studies are needed to assess their input in HDV pathogenesis.

**Acknowledgements: **This work was supported by the Russian Science Foundation (grant #19-14-0197).

### O2. Change of phenotypical characteristics of human epithelial cells made to overexpress the reverse transcriptase of HIV-1

#### Alla Kondrashova^1*^, Ekaterina Bayurova^1,2^, Darya Avdoshina^1^, Tatiana Gorodnicheva^3^, Alexander Artjukhov^3^, Erdem Dashinimayev^3^, Ilya Gordeychuk^1,2,4^, Maria G. Isaguliants^1,2,5,6^

##### ^1^Chumakov Federal Scientific Center for Research and Development of Immune-and-Biological Products of Russian Academy of Sciences, Moscow, Russia; ^2^Gamaleya National Research Center for Epidemiology and Microbiology, Moscow, Russia; ^3^Pirogov Russian National Research Medical University, Moscow, Russia; ^4^Institute for Translational Medicine and Biotechnology, Sechenov First Moscow State Medical University, Moscow, Russia; ^5^Riga Stradins University, Riga, Latvia; ^6^Department of Microbiology, Tumor and Cell Biology, Karolinska Institutet, Stockholm, Sweden

###### Presenting author: Alla Kondrashova (varyaw96@gmail.com)

*Infectious Agents and Cancer* 2022, **17(Suppl 1)**: O2

**Background: **People living with human immunodeficiency virus (HIV-1) are at increased risk of developing cancer affecting epithelial cells, even with long-term successful antiretroviral therapy, which suggests a direct tumorigenic effect of HIV proteins [1]. Earlier, we described oncogenic properties of HIV-1 reverse transcriptase (RT) [2]. The aim of this study was to evaluate the effect of stable RT expression on HPV16-infected squamous cervical cancer cells (SCCC).

**Materials and methods: **Ca Ski cells (CRL-1550) were transduced with lentiviral particles expressing HIV-1 RT under the control of phosphoglycerate kinase gene (PGK) promoter [2]. Ca Ski subclone expressing Green Fluorescent Protein (GFP) served as a control. Subclones were obtained by single cell culturing, and assessed for expression of RT by PCR and Western blotting, and for GFP, by flow cytometry. Gene inserts were confirmed by sequencing, copy number was assessed by digital drop PCR (ddPCR). ROS production was registered with 2′,7′-dichlorodihydrofluoresceine diacetate. Levels of mRNA for RT_A, HPV16 E6, E7, Nqo1, NRF2, CGCL, Nqo1, ATF4 were assessed by RT Q-PCR with SYBR Green on a RotorGene6000 cycler (Qiagen), calculated by the 2-ddCt method using GUSB for normalization [3]. Wound healing assay (WHA) was performed using Cytation 5 (BioTek) during 18 h with 1 h-interval. Data were analyzed using nonparametrical statistics (GraphPad Prism 6).

**Results: **Recombinant RT added to cultured Ca Ski increased expression of HPV 16 E6 mRNA, and induced production of ROS. Ca Ski transduced by RT-encoding lentivirus stably expressed RT after 6 month passaging, as was confirmed by RT Q-PCR and Western blotting. RT expression levels correlated with RT-gene copy number. During passaging, expression of RT mRNA by Ca Ski_RT subclones increased 3–5 times due to an increase in activity of PGK promoter overexpressed in metastatic cells [4]. On contrary to observations in Ca Ski exposed to recombinant RT, expression levels of E6 and E7 in the Ca Ski_RT subclones were stable in both short or longterm cultivation (1 and 6 month, respectively). Also, expression of RT in Ca Ski did not induce production of ROS. In lines with this, levels of transcription factors involved in cellular redox balance Nrf2 and GCLC, and of the enzyme of Phase II of oxidative stress response NQO1 mRNA remained stable over time, as in Ca Ski_GFP subclone. Compared to controls, Ca Ski_RT subclones demonstrated decreased motility, partially compensated in subclones expressing higher levels of RT. The latter demonstrated an increased capacity to heal large (> 800 micron) wounds and a loss of contact inhibition at the late stages of WHA. Expressed at low levels, RT reduced the clonogenic activity, significantly decreasing the number and size of colonies. In highly expressing Ca Ski_RT subclones the clonogenic activity was restored indicating that RT modulated the ability of cells to form colonies from single cells.

**Discussion: **We have previously shown that RT-expressing adenocarcimoma cells exhibit signs of oxidative stress, express stress-related proteins and demonstrate increased motility [2]. RT-exposed Ca Ski produced ROS, exhibited an increase in E6 expression, but had decreased motility. On contrary to this, RT-expressing Ca Ski did not produce ROS and did not express stress-related proteins. Their motility and clonogenic activity were reduced at low RT levels, but resqued in highly RT-expressing Ca Ski subclones.

**Conclusions: **Difference in transient and long-term effects of RT could be explained by the capacity of HPV16-infected SCCC to suppress RT-induced production of ROS and downstream events. The mechanism of resque of motility and clonogenic activity of SCCC by high levels of HIV-1 RT needs further investigation.

**Acknowledgements: **RFBR 19_04_01034.

**References**

1. Bayurova E, Jansons J, Skrastina D, Smirnova O, Mezale D, Kostyusheva A, Kostyushev D, Petkov S, Podschwadt P, Valuev-Elliston V, Sasinovich S, Korolev S, Warholm P, Latanova A, Starodubova E, Tukhvatulin A, Latyshev O, Selimov R, Metalnikov P, Isaguliants, M. HIV-1 Reverse Transcriptase Promotes Tumor Growth and Metastasis Formation via ROS-Dependent Upregulation of Twist. Oxidative Medicine and Cellular Longevity, 2019, 1–28. https://doi.org/10.1155/2019/6016278

2. Isaguliants, M., Bayurova, E., Avdoshina, D., Kondrashova, A., Chiodi, F., & Palefsky, J. M. Oncogenic Effects of HIV-1 Proteins, Mechanisms Behind. Cancers, 2021, 13(2). https://doi.org/10.3390/cancers13020305

3. Zhang J.D., Ruschhaupt M., Biczok R. ddCt Method for qRT–PCR Data Analysis. http://bioconductor.jp/packages/2.14/bioc/vignettes/ddCt/inst/doc/rtPCR.pdf 2010. Accessed Aug. 2020.

4. Ahmad SS, Glatzle J, Bajaeifer K, Bühler S, Lehmann T, Königsrainer I, Vollmer JP, Sipos B, Ahmad SS, Northoff H, Königsrainer A, Zieker D. Phosphoglycerate kinase 1 as a promoter of metastasis in colon cancer. Int J Oncol. 2013 Aug;43(2):586–90. https://doi.org/10.3892/ijo.2013.1971.

### O3. Trans cohorts metabolic reprogramming towards glutaminolysis in long-term successfully treated HIV-1 infection

#### Flora Mikaeloff^1*^, Sara Svensson-Akusjärvi^1^, George Mondinde Ikomey^2^, Soham Gupta^1^, Alejandra Escós^1^, Luke Elizabeth Hanna^3^, Kamal Singh^4^, Rui Benfeitas^5^, Ujjwal Neogi^1,4,6^

##### ^1^Karolinska Institute, Stockholm, Sweden; ^2^University of Yaoundé 1, Yaoundé, Cameroon; ^3^National Institute for Research in Tuberculosis, Chennai, India; ^4^University of Missouri, Columbia, USA; ^5^Stockholm University, Stockholm, Sweden; ^6^Manipal Academy of Higher Education, Manipal, Karnataka, India

###### Presenting author: Flora Mikaeloff (flora.mikaeloff@ki.se)

*Infectious Agents and Cancer* 2022, **17(Suppl 1)**: O3

**Background: **Despite successful combination antiretroviral therapy (cART), persistent low-grade immune activation together with inflammation and toxic antiretroviral drugs can lead to long-lasting metabolic disruption and adaptation in people living with HIV (PLWH)1, 2, 3. Our study investigated alterations in the plasma metabolic profiles by comparing PLWH on long-term cART(> 5 years) and matched HIV-negative controls (HC) in two cohorts from low- and middle-income countries (LMIC), Cameroon, and India, respectively, to understand the system-level dysregulation in HIV-1 infection. We also characterized alterations in cellular metabolism during the steady-state of latent HIV infection using quantitative proteomics analysis in pro-monocytic latent cell model.

**Materials and methods: **Plasma samples from 138 PLWH on cART, 126 HC, and 45 untreated HIV-infected patients with viremia from Cameroon (n = 171) and India (n = 138) were sent for untargeted and targeted LC–MS/MS-based metabolic profiling. We applied three methodologies: non-parametric Mann–Whitney U test, Partial Least Squares Discriminant Analysis (PLS-DA) and random Forest (RF) to find biomarkers differing HC and PLWH in untargeted data independently in India and Cameroon cohorts. We performed metabolite set enrichment to find HIV-specific pathways. We confirmed untargeted metabolomics results using targeted metabolomics. We performed quantitative proteomic analysis using LC–MS/MS in pro-monocytic latent cell model U1, and lymphocytic latent cell model J-lat10.6 together with their respective uninfected parental cell lines, U937 and Jurkat. Differential abundance analysis was performed using R package LIMMA.

**Results: **An altered amino acid metabolism, more specifically to glutaminolysis in PLWH than HC was reported from analysis of untargeted and targeted metabolomics. A significantly lower level of neurosteroids was observed in both cohorts and could potentiate neurological impairments in PLWH. Further, modulation of cellular glutaminolysis promoted increased cell death and latency reversal in pre-monocytic HIV-1 latent cell model U1, which may be essential for the clearance of the inducible reservoir in HIV-integrated cells.

**Conclusions: **In conclusion, our present study based on two cohorts indicated altered AA metabolism and more potentially a switch in glutaminolysis as the alternative pathway for energy production following a long-term antiretroviral therapy.

**References**

1. Babu H, et al. Plasma Metabolic Signature and Abnormalities in HIV-Infected Individuals on Long-Term Successful Antiretroviral Therapy 2019; Metabolites 9.

2. Babu H, et al. Systemic Inflammation and the Increased Risk of Inflamm-Aging and Age-Associated Diseases in People Living With HIV on Long Term Suppressive Antiretroviral Therapy. Front Immunol (2019) 10, 1965.

3. Gelpi M, et al. Central role of the glutamate metabolism in long-term antiretroviral treated HIV-infected individuals with metabolic syndrome: a cross-sectional cohort study. medRxiv, 2021.2004.2001.21254778.

### O4. Expression of HIV-1 reverse transcriptase in tumor cells of epithelial origin modulates their mitochondrial respiration and motility

#### Ekaterina Bayurova^1,2*^, Alla Kondrashova^1^, Natalia F. Zakirova^3^, Darya Avdoshina^1^, Tatiana Gorodnicheva^4^, Juris Jansons^5,6^, Dace Skrastina^5^, Svetlana Gebrila^6,7^, Alecia Dudorova^6,7^, Jurijs Nazarovs^7^, Ilya Gordeychuk^1, 2,8^, Alexander V. Ivanov^2,3^, Maria G. Isaguliants^1,2,6^

##### ^1^Chumakov Federal Scientific Center for Research and Development of Immune-and-Biological Products of Russian Academy of Sciences, Moscow, Russia; ^2^Gamaleya National Research Center for Epidemiology and Microbiology, Moscow, Russia; ^3^Engelhardt Institute of Molecular Biology, Moscow, Russia; ^4^Evrogen, Moscow, Russia; ^5^Latvian Biomedical Research and Study Centre, Riga, Latvia; ^6^Riga Stradins University, Riga, Latvia; ^7^Paul Stradins University Hospital, Riga, Latvia; ^8^Institute for Translational Medicine and Biotechnology, Sechenov First Moscow State Medical University, Moscow, Russia

###### Presenting author: Ekaterina Bayurova (bayurova_eo@chumakovs.su)

*Infectious Agents and Cancer* 2022, **17(Suppl 1)**: O4.

**Background: **HIV-1 infected people are characterized by high prevalence of cancers affecting epithelial cells despite successful antiretroviral treatment. We hypothesized that this is due to the direct carcinogenic properties of HIV-1 proteins, including reverse transcriptase (RT) [1]. The aim of this study was to dissect the mechanism(s) underlying RT carcinogenicity by evaluating the effect of HIV RT expression on the metabolic activity and motility of tumor cells of epithelial origin.

**Materials and methods: **RT_A expressing subclones of 4T1luc2 cells were described previously [2]. Ca Ski cells (CRL-1550) were transduced with lentiviral particles expressing RT of HIV clade A FSU_A strain RT_A described earlier [2]. Resulting cells were cloned by single cell culturing, subclones were assessed for RT expression by PCR and Western blotting. ROS production was registered with 2′,7′-dichlorodihydrofluoresceine diacetate. Expression of *E-cadherin, N-cadherin, Vimentin, Twist*, *Snail*, and *tubulin A and G* was assessed by RT Q-PCR with SYBR Green on a RotorGene6000 cycler (Qiagen). Wound healing assay (WHA) was performed using Cytation 5 (BioTek) for 18 h with 1 h interval. Mitochondrial respiration and glycolysis were assessed using Seahorse technology (Agilent). Data were analyzed using nonparametrical statistics (GraphPad Prism 6), p < 0.05 was assigned significant.

**Results: **In mammary gland adenocarcinoma 4T1 cells, RT expression led to increased production of ROS, enhanced cell motility, and overexpression of mRNA of EMT factors including Twist, dependent on the levels of RT expression. In metabolic assays, RT-expressing 4T1 cells demonstrated enhanced basal respiration and spare respiratory capacity with no significant changes in glycolysis. Implanted into BALB/C mice, RT-expressing 4T1 cells caused faster tumor growth and higher metastasic activity than parental 4T1 cells (p < 0.05). Tumor size and metastatic activity were proportional to RT expression. In cervical cancer Ca Ski cells, stable expression of RT led to suppressed basal respiration and decreased respiratory capacity, increased glycolysis, and at the same time, decreased the expression of mRNA of Twist, Nrf2, and tubulins A and G, and reduced directional cell motility. At the same time, RT-expressing Ca Ski demonstrated increased capacity to heal large wounds and loss of contact inhibition at the late stages of WHA, both characteristics of respiration and motility consistent with the distruction of microtubuli.

**Conclusions: **Expression of HIV-1 RT causes changes in mitochondrial respiration. Change in respiration parameters varies depending on the type of expressing cells, with enhancement of respiration and cell motility in epithelial breast cancer, and suppression of respiratory capacity, basal cell respiration and cell motility in cervical cancer cells. Fine interplay exists between respiration, mitochondrial morphology, and microtubule and microfilament polymerization, reduced basal respiration arising as a symptom of cytoskeletal impairment [3]. Cumulatively, our data indicate that HIV-1 RT is heavily involved in this interplay. Differential outcome of this involvement may be due to differences in the functions of microtubuli and regulation of mitochondrial respiration in the breast cancer cells, which are metabolically very plastic [4], compared to HPV-infected cervical cancer cells with a typical (more rigid) cancer metabolic profile [5].

**Acknowledgements: **Russian Fund for Basic Research 19_04_01034; Russian Science Foundation grant 19-74-10086.

**References**

1. Isaguliants M, Bayurova E, Avdoshina D, Kondrashova A, Chiodi F, Palefsky JM. Oncogenic Effects of HIV-1 Proteins, Mechanisms Behind. Cancers (Basel). 2021 Jan 15;13(2):305. https://doi.org/10.3390/cancers13020305

2. Bayurova E, Jansons J, Skrastina D, Smirnova O, Mezale D, Kostyusheva A, Kostyushev D, Petkov S, Podschwadt P, Valuev-Elliston V, Sasinovich S, Korolev S, Warholm P, Latanova A, Starodubova E, Tukhvatulin A, Latyshev O, Selimov R, Metalnikov P, Komarov A, Ivanova O, Gorodnicheva T, Kochetkov S, Gottikh M, Strumfa I, Ivanov A, Gordeychuk I, Isaguliants M. HIV-1 Reverse Transcriptase Promotes Tumor Growth and Metastasis Formation via ROS-Dependent Upregulation of Twist. Oxid Med Cell Longev. 2019 Dec 2;2019:6016278. https://doi.org/10.1155/2019/6016278.

3. Kandel J, Angelin AA, Wallace DC, Eckmann DM. Mitochondrial respiration is sensitive to cytoarchitectural breakdown. Integr Biol (Camb). 2016 Nov 7;8(11):1170–1182. https://doi.org/10.1039/c6ib00192k.

4. Avagliano A, Ruocco MR, Aliotta F, et al. Mitochondrial Flexibility of Breast Cancers: A Growth Advantage and a Therapeutic Opportunity. Cells. 2019;8(5):401. Published 2019 Apr 30. https://doi.org/10.3390/cells8050401

5. Li B, Sui L. Metabolic reprogramming in cervical cancer and metabolomics perspectives. Nutr Metab (Lond). 2021 Oct 19;18(1):93. https://doi.org/10.1186/s12986-021-00615-7.

### O5. Metabolic changes in hepatocytes during HCV infection: a study in the physiological media

#### Michail V. Golikov^1^*, Natalia F. Zakirova^1^, Olga N. Ivanova^1^, Irina T. Fedyakina^2^, Birke Bartosch^3^, Olga A. Smirnova^1^, Alexander V. Ivanov^1^

##### ^1^Engelhardt Institute of Molecular Biology, Russian Academy of Sciences; ^2^Gamaleya National Research Centre for Epidemiology and Microbiology of the Ministry of Russia, Moscow, Russia; ^3^University of Lyon, Université Claude Bernard Lyon 1, INSERM 1052, CNRS 5286, Centre Léon Bérard, Centre de recherche en cancérologie de Lyon, Lyon, France

###### Presenting author: Michail V. Golikov (cool.mik3492594@yandex.ru)

*Infectious Agents and Cancer* 2022, **17(Suppl 1)**: O5

**Background: **Hepatitis C virus (HCV) is one of the major pathogens causing chronic liver disease. Chronic hepatitis C patients may have a variety of liver pathologies, including fibrosis, cirrhosis, steatosis, hepatocellular carcinoma, etc. Various virus-associated pathologies are dependent on metabolic changes that the virus causes in the host cell. It is also known that the metabolism of a cell strongly depends on the conditions of cultivation (including the growth medium). Studies of hepatocyte metabolism during HCV infection may have significantly distorted results due to too strong influence of culture media (e.g. MEM, DMEM), which were created for long-term maintenance of cell cultures and do not reflect real physiological conditions. In recent years, physiologically based mediums (e.g. Plasmax, HPLM) have been proposed and shown that metabolic changes in such conditions often have a fundamentally different effect [1–3]. The aim of this study was to dissect the metabolic changes in hepatocytes and other cell lines in response to Plasmax medium and to investigate replication of various RNA viruses in cells cultivated in Plasmax.

**Materials and methods: **We evaluated impact of Plasmax medium on cell metabolism using Seahorse technology, visualization of mitochondria by confocal microscopy, analysis of ROS production and mitochondrial mass by flow cytometry in four different mammalian cell lines, alongside with medium effects on replication of eight different viruses.

**Results: **Plasmax medium increased basal mitochondrial respiration and spare respiratory capacity in all four cell lines. It was accompanied by assembly of mitochondria into vast networks without changes in mitochondrial mass. Noteworthy that Plasmax significantly reduced lysosomal mass in all cell lines. In lines with this, the cells were much more sensitive to inhibitors of respiratory complex II and ATP synthase as well as to an inhibitor of fatty acid catabolism pointing to increased input of this pathway into central carbon metabolism. Plasmax medium also suppressed replication of various RNA viruses including HCV, influenza A virus and SARS-CoV-2. Nevertheless, all these viruses in cells cultivated in Plasmax triggered oxidative stress. qPCR analysis in HCV-infected Huh7.5 cells identified changes in expression of various metabolic genes.

**Conclusions: **Studies of metabolic changes in virus-infected cells, especially of those concerning mitochondria, lysosomes, or redox systems, should be performed in Plasmax or HPLM medium.

**Acknowledgements: **The study was supported by the Russian Science Foundation grant No. 19-74-10086.

**References**

1. J. R. Cantor et al., “Physiologic Medium Rewires Cellular Metabolism and Reveals Uric Acid as an Endogenous Inhibitor of UMP Synthase,” Cell, vol. 169, no. 2, pp. 258–272.e17, 2017.

2. J. Vande Voorde et al., “Improving the metabolic fidelity of cancer models with a physiological cell culture medium,” Sci. Adv., vol. 5, no. 1, 2019. 4.

3. Cantor J.R., “The Rise of Physiologic Media,” Trends Cell Biol., vol. 29, no. 11, pp. 854–861, 2019.

## Session “chronic viral infections and cancer”

### PL4. Reversible and irreversible activation of telomerases in HPV-related cancers

#### Maria Lina Tornesello^1*^, Noemy Starita^1^, Andrea Cerasuolo^1^, Anna Lucia Tornesello^1^, Franco Ionna^2^, Cono Scaffa^3^, Stefano Greggi^3^, Gabriella Aquino^4^, Simona Losito^4^, Franco Maria Buonaguro^1^

##### ^1^Molecular Biology and Viral Oncology Unit, Istituto Nazionale Tumori IRCCS "Fond. Pascale"; ^2^Maxillofacial and Ear Nose and Throat Surgery Dept, Istituto Nazionale Tumori IRCCS "Fond Pascale"; ^3^Gynaecology Oncology Unit, Istituto Nazionale Tumori IRCCS "Fondazione G. Pascale"; ^4^Department of Pathology, Istituto Nazionale Tumori IRCCS "Fondazione G. Pascale", Naples, Italy

###### Presenting author: Maria Lina Tornesello (m.tornesello@istitutotumori.na.it)

*Infectious Agents and Cancer* 2022, **17(Suppl 1)**: PL4

**Background: **Human telomerase reverse transcriptase (hTERT or TERT) is the catalytic subunit of the telomerase enzyme, which elongates telomeres at the ends of chromosomes during embryonic development and in stem cells. TERT gene is repressed in somatic cells and telomeres undergo progressive shortening at each division (Hayflick limit) until senescence. Nevertheless, telomerase is re-expressed in up to 90% of human cancers through several mechanisms, including somatic mutations, genetic and epigenetic alterations and virus integration. In HPV-related cancers, TERT gene is always expressed in cells infected by high risk HPVs at diverse body sites, such as cervix, vulva, vagina, anus, penis and head-and-neck cancers. Several studies have shown that high risk HPV E6 oncoprotein is a main regulator of TERT expression for its ability to transactivate the TERT promoter. Nevertheless, in cervical neoplasia, telomerase expression was found to be significantly correlated with the histological severity of cervical lesions regardless of HPV E6 RNA levels.

**Results: **An HPV-independent activation of telomerase is caused by hot spot changes at position -124 (G > A) and -146 (G > A) in the core promoter region of the TERT gene. We have identifed, by Sanger sequencing and droplet digital PCR, the TERT promoter mutations -124A and -146A in 17% of cervical, 53% of vulvar and 54% of penile squamous cell carcinoma (SCC) as well as in 60% of oral carcinoma. Overall TERT promoter mutations were more frequent in tumours negative for HPV infection. TERT promoter mutations were not detected in cervical intraepithelial neoplasia grade 3 (CIN3). In HPV-positive cervical cancer cases TERT gene expression was significantly higher in TERT promoter mutated than in non-mutated tumours regardless of HPV16 E6 levels. The expression profile of p53-related genes showed that telomerase overexpression driven by promoter mutations is associated with the activation of extratelomeric functions involving among others the IGF1R/AKT signalling axis in cervical SCC as well as in TERT promoter mutated SiHa cell line.

**Discussion and conclusions: **Our results suggest that in cervical neoplasia the promoter mutations have a much stronger effect in TERT activation than HPV E6 oncoprotein. We can hypothesize that the role of telomerase in HPV-related cancers is dual: (1) telomerase expression is reversibly regulated by viral E6 protein in the early stages of tumorigenesis, and (2) telomerase is irreversibly and highly activated by genetic alterations and promoter mutations in TERT gene in progressing cervical neoplasia. The knowledge of non-canonical functions activated by highly expressed telomerase would be valuable to identify new actionable targets in SCC of the lower genital tract as well as in many TERT mutated tumours.

**Acknowledgements: **This work was supported by research grants funded by Ricerca Corrente (M1-3) and Ministero della Salute (RF-2018-12366163). We thank Vincenzo Gigantino for assistance in sample selection, retrieval and experimental procedures.

**References**

1. Annunziata C, Pezzuto F, Greggi S, Ionna F, Losito S, Botti G, Buonaguro L, Buonaguro FM, Tornesello ML. Distinct profiles of TERT promoter mutations and telomerase expression in head and neck cancer and cervical carcinoma. Int J Cancer. 2018 Sep 1;143(5):1153–1161. https://doi.org/10.1002/ijc.31412.

2. Tornesello ML, Buonaguro FM. Human Papillomavirus and Cancers. Cancers (Basel). 2020 Dec 15;12(12):3772. https://doi.org/10.3390/cancers12123772.

3. Cerasuolo A, Annunziata C, Tortora M, Starita N, Stellato G, Greggi S, Maglione MG, Ionna F, Losito S, Botti G, Buonaguro L, Buonaguro FM, Tornesello ML. Comparative analysis of HPV16 gene expression profiles in cervical and in oropharyngeal squamous cell carcinoma. Oncotarget. 2017 May 23;8(21):34070–34081. https://doi.org/10.18632/oncotarget.15977.

### PL5. Chronic inflammation in HIV-1 infection, are elite controllers different?

#### Nicolas Noel*

##### Service de Médecine Interne et Immunologie Clinique, GHU Paris Saclay, AP-HP BICÊTRE, Faculté de Médecine Paris Saclay; INSERM /CEA 1184 (Immunologie des maladies virales, autoimmunes, bactériennes et hématologiques);

###### Presenting author: Nicolas Noel (nicolas.noel@aphp.fr)

*Infectious Agents and Cancer* 2022, **17(Suppl 1)**: PL5

The Human Immunodeficiency Virus is responsible for a chronic viral infection during which antiretroviral therapy is usually mandatory to maintain an undetectable viremia and to preserve immune functions.

Immune activation is a hallmark of this pathology, occurring in the early stages of infection. Multiple sources of immune activation have been described: viral replication and production, antigen presentation, microbial translocation of mucosal origin, activation of the interferon pathway, viral co-infections, etc. Moreover, immune activation is linked with the control of the HIV reservoir and viral latency, as well as with the risk of clinical events such as cardiovascular or neurocognitive diseases.

Antiretroviral treatments reduce chronic immune activation but not always normalize inflammatory parameters. In this context, HIV-1 controllers allow an interesting study of the causes and consequences of inflammation. These rare patients are defined on a virological basis, controlling viral replication spontaneously without any antiretroviral treatment. This lecture will present the evidence of persistent immune activation in HIV-controller patients in comparison with controlled patients under treatment, and discuss the risk of evolution and the research perspectives in this field.

### PL6. Mechanisms of nucleic acid vaccines for therapy of chronic viral infections and cancer

#### Margaret A. Liu ^1,2,3^

##### ^1^ProTherImmune, Lafayette, CA, USA; ^2^Karolinska Institutet, Stockholm, Sweden; ^3^University of California, San Francisco, USA

##### Presenting author: Margaret A. Liu (Liu@ProTherImmune.com)

*Infectious Agents and Cancer* 2022, **17(Suppl 1)**: PL6

Immunotherapies for chronic viral infections and cancer have utilized approaches including monoclonal and bispecific antibodies, immunomodulatory agents (cytokines and checkpoint inhibitors), vaccines (utilizing various delivery systems), and adoptive cell therapies. The efficacy of certain antibodies, immunomodulators, and cell therapies for certain cancers have demonstrated their potential. This has led to increased efforts to develop immunotherapeutic vaccines targeting tumor and viral antigens. But challenges remain even after selecting an appropriate antigen to target, whether a viral protein or a tumor antigen. One reason for this is that tumors and chronic infections can affect the immunological milieu resulting in tolerance or immune suppression. This talk will describe the use of nucleic acid vaccines as potential vectors for therapeutic vaccines for both chronic infections and cancer. The focus will be on the immune mechanisms stimulated by these vaccines, including innate immunity and how these innate immune responses may contribute to both the specific immune responses against an encoded antigen as well as for non-specific immune benefits including immune fitness.

### PL7. The path towards the cure of chronic HBV infection

#### Fabien Zoulim^1,2^

##### ^1^Hepatology Department, Hospices Civils de Lyon; ^2^Cancer Research Center of Lyon, Lyon, France

###### Presenting author: Fabien Zoulim (fabien.zoulim@inserm.fr)

*Infectious Agents and Cancer* 2022, **17(Suppl 1)**: PL7

Hepatitis B virus (HBV) affects more than 250 million people worldwide, and is one of the major aetiologies for the development of cirrhosis and hepatocellular carcinoma (HCC). In spite of universal vaccination programs, HBV infection is still a public health problem, and the limited number of available therapeutic approaches complicates the clinical management of these patients. Thus, HBV infection remains an unmet medical need that requires a continuous effort to develop new individual molecules, treatment combinations and even completely novel therapeutic strategies to achieve the goal of HBV elimination. Progress in our understanding of HBV persistence and pathobiology have allowed to characterize novel antiviral and immune targets for therapy, as well as novel biomarkers to assess the intrahepatic viral reservoir and host antiviral immune responses. These discoveries are now translated to chronic hepatitis B patients with clinical trials of combination therapy with direct acting antivirals and immune modulators. These clinical and translational studies show promising results that may pave the way for a functional cure of HBV and the prevention of HCC development. It will be exciting to see how the drug discovery and biomarker pipelines will feed the clinical development of novel treatments and guide clinical investigations to treat all facets of the disease.

### O6. Activity of long control region of human papilloma virus as determinant of oncogenicity

#### Riaz Y. Seedat^1^, Catharina E. Combrinck^2^, Yuri Munsamy^2^, Felicity J. Burt^2,3*^

##### ^1^Department of Otorhinolaryngology, University of the Free State, Bloemfontein, South Africa; ^2^Division of Virology, University of the Free State, Bloemfontein, South Africa; ^3^National Health Laboratory Service Universitas, Bloemfontein, South Africa

###### Presenting author: Felicity J. Burt (burtfj@ufs.ac.za)

*Infectious Agents and Cancer* 2022, **17(Suppl 1)**: O6

**Background: **Human papilloma viruses (HPV) have been associated with benign and malignant diseases. Approximately 150 HPV genotypes have been described, including high risk or carcinogenic viruses such as HPV 16 and 18 associated with cervical cancers, high risk HPV 31 associated with head and neck squamous cell carcinoma, and low risk types such as 6 and 11 more frequently associated with benign lesions and are the aetiological agents of recurrent respiratory papillomatosis (RRP). The HPV genome is organized into early (E) and late (L) open reading frames (ORFs), based on the position of these genes on the genome, and includes a noncoding region, or long control region (LCR) comprising crucial elements necessary for control of viral replication and transcription as well as the origin of DNA replication. The influence of mutations within the LCR is not clear and warrants investigation. The aim of this study was to use a reporter gene system to determine if mutations identified in the non-coding LCR of two HPV clinical isolates (type HPV 31 and HPV 6) had an influence on transcriptional activity.

**Methods: **Mutations in the LCR were identified for two HPV isolates during phylogenetic studies. The complete genome of an HPV isolate from a laryngeal squamous cell carcinoma was determined with sequence variations relative to the HPV 31 prototype. The complete genome of an HPV 6 isolate from a patient with aggressive RRP was determined and a 170 bp duplication was detected in the LCR. In each instance the non-coding region was amplified and cloned upstream of a heterologous reporter gene and the activity of the reporter gene product determined using transfected cells.

**Results: **Reporter gene activity from an HPV 6 isolate derived LCR region with a 170 bp duplication was significantly higher than a control with no duplication. Similarly enhanced transcriptional activity was observed for a reporter gene system constructed using HPV 31 derived LCR with nucleotide variations in the p97 promotor region. Enhanced transcriptional activity was observed with the mutant that possessed a single nucleotide change within the YY1 transcription binding site.

**Conclusion: **Sequence variation within the LCR may have a functional effect on viral promotor activity. The results suggest that HPV isolates with mutations in the non-coding region warrant further investigation for potential biomarkers of aggressive disease.

**Acknowledgements: **Research was funded by the National Research Foundation. South Africa Society of Otorhinolaryngology Head and Neck Surgery Research Fund.

### O7. Comparative characteristics of TC-1 and novel 4T1-based cell lines expressing HPV 16 oncoproteins E6 and E7 in ability to reproduce HPV-associated carcinogenesis in a mouse model

#### Darya Avdoshina^1*^, Alesja Dudorova^2,3*^, Svetlana Gebrila^2^, Alla Kondrashova^1^, Ekaterina Bayurova^1,4^, Tatiana Gorodnicheva^5^, Olga A. Kuznetsova^6^, Ekaterina M. Olyushina^6^, Alexander Artyukhov^7^, Erdem Dashinimaev^7^, Dmitry Kostyshev^8,9^, Anastasia Kostysheva^8^, Amir I. Tukhvatulin^4^, Dace Skrastina^10^, Sergejs Isajevs^11,12^, Juris Jansons^2,10^, Ilya Gordeychuk^1,4,13^, Maria G. Isaguliants^2,4,14^

##### ^1^Chumakov Federal Scientific Center for Research and Development of Immune-and-Biological Products of Russian Academy of Sciences, Moscow, Russia; ^2^Riga Stradins University, Riga, Latvia; ^3^Paul Stradins University Hospital, Riga, Latvia; ^4^NF Gamaleya Research Center of Epidemiology and Microbiology, Moscow, Russia; ^5^Evrogen, Moscow, Russia; ^6^Medical Academy for Continuous Professional Education, Moscow, Russia; ^7^Center for Precision Genome Editing and Genetic Technologies, Pirogov Russian National Research Medical University, Moscow 127994, Russia; ^8^National Medical Research Center for Tuberculosis and Infectious Diseases, Moscow, Russia; ^9^Scientific Center for Genetics and Life Sciences, Division of Biotechnology, Sirius University of Science and Technology, 354340 Sochi, Russia; ^10^Latvian Biomedical Research and Study Center, Riga, Latvia; ^11^Department of Pathology, Faculty of Medicine, University of Latvia, Riga, Latvia; ^12^Centre of Pathology, Riga East University Hospital, Riga, Latvia; ^13^Sechenov First Moscow State Medical University, Moscow, Russia; ^14^Peoples Friendship University of Russia (RUDN University), Moscow, Russia

###### Presenting authors: shared first co-authorship Darya Avdoshina (avdoshina_dv@chumakovs.su), Alesja Dudorova (alesja.dudorova@rsu.lv)

*Infectious Agents and Cancer* 2022, **17(Suppl 1)**: O7

**Background: **Prophylactic HPV vaccination prevents nearly 90% of infections and HPV-associated neoplasia, however, vaccine coverage is insufficient. Besides, prophylactic vaccination does not clear HPV post-infection, requesting development of therapeutic HPV vaccines to cure chronic infections and associated cancerous lesions. Such development relies on small animal models. A widely used murine model today is based on primary lung epithelial cells co-transformed with the HPV16 E6/E7 and activated c-H-Ras TC-1 [1] and its subclones carrying Luciferase (Luc), syngenic to C57Bl/6 mice. Models in other mouse strains are few, and are costly to establish and use [2, 3]. The aim of the study was to create model of HPV-associated cancer syngenic to BALB/c mice based on murine mammary gland adenocarcinoma 4T1luc2 cells, compare their in vitro and in vivo properties with established TC-1 model.

**Material and methods: **DNA encoding E6 and E7 from HPV16 carrying plasmid (ATCC) was recloned into lentiviral vector pLJM1 (Addgene). Resulting lentivirus was used to transduce 4T1luc2 cells (“Bioware Ultra Cell Line 4T1luc2,” Caliper) generating subclones overexpressing E6/E7 of HPV 16 (n = 9). Cell line TC-1/luc2 was kindly donated by Prof TC Wu (John Hopkins University, USA). Expression of E6/E7 mRNA was assessed by real-time RT-PCR. Genetic stability of cells was assessed by counting γ-H2AX foci after staining with anti-γ-H2AX MAb26350 (Abcam) Alexa Fluor594 anti-mouse Ab150116 (Abcam) and nuclear staining with Hoechst. Cell cycle analysis after propidium iodide staining was performed on BD FACSCantoII cytometer (BD Biosciences). 4T1 subclones were ectopically implanted into BALB/c and TC-1, into C57bl/6 mice; tumor growth was monitored by in vivo, and metastatic activity, by ex vivo (organ) bioluminescence imaging (BLI) (Lumina, PerkinElmer) as described [4, 5]. Formalin-fixed paraffin-embedded (FFPE) tumors and organs were prepared [4, 5] to grade tumors, and assess metastatic activity. For this, FFPE blocks were sectioned, stained with H&E, scanned on digital scanner (Leica). Number and size of metastases were quantified using ImageScope software (Leica, Germany). Data were analysed using nonparametrical statistics (STATISTICA 11, Tibco; Graphpad); *p* values < 0.05 were considered significant.

**Results: **TC-1 cells carry > 500 E6/E7 gene copies [1], while 4T1luc2-based subclones had < 1. All 4T1 subclones expressed E6/E7 mRNA, levels of E6 and E7 mRNA were correlated (r = 0.9, p < 0.005). Expression of E6/E7 mRNA in 4T1luc2 induced expression of TERT (for E6 r = 0.8, for E7 r = 0.7, p < 0.05). E6/E7-expressing 4T1luc2 demonstrated signs of G0/G1 arrest, with increase in G0/G1 population inversely correlated to population in S-phase (r = -0.8, p = 0.005), which was in turn correlated to E7 mRNA levels. For TC-1, on the contrary, cells accumulated in S-phase, accumulation positively correlated with expression of E7 mRNA. Both TC-1 and E6/E7-expressing 4T1luc2 subclones exhibited γ-H2AX foci indicating dsDNA damage attributed to production of ROS. Implanted into mice, both TC-1 and E6/E7-expressing 4T1luc2 cells formed solid tumors of similar size and growth rate (p > 0.1). Ex vivo BLI assessment of infiltration of tumor cells into organs demonstrated preference of lungs in 4T1- and of spleen in TC-1 models, with similar levels of infiltration into the liver. TC-1 and E6/E7-expressing 4T1luc2 cells did not differ in the number and size of liver metastases, both were inferior to the parental 4T1luc2 cells (p < 0.05). Size and number of metastases caused by implanted E6/E7 expressing 4T1luc subclones did not correlated with the level of expression of E6/E7 mRNA.

**Conclusions: **Parameters of tumor growth and metastatic activity of E6/E7-expressing 4Tluc2 cells were similar to TC-1. E6/E7 expression in 4T1 cells did not cause enhanced tumor growth, or increased metastatic activity, although the main affected organ were the lungs, not the spleen as in the TC-1 model. Interestingly, in 4T1luc2 cells, as in squamous cell carcinomas in humans, expression of E6/E7 caused overexpression of TERT. New subclones could be useful for testing HPV vaccines in BALB/c mice.

**Acknowledgements: **LZP-2018/2-0308 to M.I., LZP2020/2-0376 to A.D., J.J. and M.I., NAWA to M.I., RFBR 20-015-00442 to D.K., A.K, and RFBR 19-04-01034.

**References**

1. Lin K.Y. et al., *Cancer Res 1996,* **56, **21–26.pmid:8548765.

2. Doorbar J. J Pathol. 2016 Jan;238(2):166–79. https://doi.org/10.1002/path.4656.

3. Henkle T.R. et al. Cancer Res. 2021 Sep 1;81(17):4560–4569. https://doi.org/10.1158/0008-5472.CAN-21-0399.

4. Bayurova E. et al., Oxid Med Cell Longev. 2019 Dec 2;2019:6016278. https://doi.org/10.1155/2019/6016278.

5. Jansons J. et al., Vaccines (Basel). 2020 Jun 18;8(2):318. https://doi.org/10.3390/vaccines8020318.

### O8. Ongoing hepatitis D virus epidemics in Tuva despite 20 years of vaccination against hepatitis B

#### Karen K. Kyuregyan^1,2,3*^, Anastasiya A. Karlsen^1,2,3^, Olga V. Isaeva^1,2^, Oleg V. Kuzmin^2^, Ilya A. Potemkin^1,2^, Fedor A. Asadi Mobarhan^2^, Eugeniy V. Mullin^2^, Victor A. Manuylov^4^, Andrei A. Pochtovyy^4^, Vladimir A. Gushchin^4^, Anna A. Saryglar^5^, Lyudmila Yu. Ilchenko^2^, Mikhail I. Mikhailov^1,2^

##### ^1^Russian Medical Academy of Continuous Professional Education, Moscow, Russia; ^2^Mechnikov Research Institute of Vaccines and Sera, Moscow, Russia; ^3^Peoples' Friendship University of Russia (RUDN University), Moscow, Russia; ^4^N.F. Gamaleya National Research Center for Epidemiology and Microbiology, Moscow, Russia; ^5^Kyzyl Hospital of Infectious Diseases, Kyzyl, Russia

###### Presenting author: Karen K. Kyuregyan (karen-kyuregyan@yandex.ru)

*Infectious Agents and Cancer* 2022, **17(Suppl 1)**: O8

**Background: **Hepatitis delta virus (HDV) causes severe rapidly progressive chronic hepatitis in patients co-infected with hepatitis B virus (HBV). HDV infection can be indirectly prevented by vaccination against HBV. Due to this, HDV is believed to be a vanishing infection in countries with successful HBV vaccination programs. We aimed to estimate evolutionary and epidemiological dynamics of HBV and HDV in Tuva, the region of the Russian Federation that has been highly endemic for HBV in pre-vaccination era and had one of the highest rates of liver cirrhosis and liver cancer among Russian regions in recent years.

**Materials and methods: **Time-scaled phylogeographic Bayesian analysis was performed separately for 73 HDV complete genome sequences obtained in Tuva in 2017–2019 and for 64 HBV sequences presenting the 676 nt long fragment of S-gene collected in Tuva in 2008–2009 supplemented with 25 HBV sequences obtained in 2021. The Skyline methods were used to extract data on past population dynamics of HBV and HDV in Tuva from phylogenetic trees.

Sera of 1170 healthy volunteers from Tuva collected in 2019, i.e., 22 years after the start of the universal HBV vaccination of newborns in the region, were tested for HBsAg and anti-HDV using commercially available ELISA kits (Vector-Best, Novosibirsk, Russia). Ten age groups were enrolled: less than 1 year, 1–4 years, 5–9 years, 10–14 years, 15–19 years, 20–29 years, 30–39 years, 40–49 years, 50–59 years, and over 60 years; each age group included roughly 123 people.

**Results: **Bayesian analysis has shown that HBV had a long history of circulation in Tuva with the time to most common ancestor (MRCA) for predominant genotype HBV-D dated back to 972 (95% HPD: 535-1253) for subtype D1, 1274 (95% HPD: 936-1384) for D2 and 1173 (95% HPD: 1005-1618) for D3. However, HDV circulation in Tuva started much later and resulted from two waves of introduction of HDV genotype 1 (HDV-1). First wave was associated with HDV-1 sequences from Central Asia and its estimated TMCA is dated 1811 (95% HPD: 1741–1834). The second wave of HDV-1 introduction was associated with strains from Russia, its TMCA is dated in 1960s (95% HPD: 1953–1979). SkyGrid reconstruction of population dynamics showed an increase in the intensity of the spread of HDV since the 1990s with its peak in the 2010s. The reproduction number (Re) for HDV in Tuva calculated based on the population dynamics predicted using Birth–Death Skyline analysis increased rapidly after 2010s, reaching the plateau of about 3 cases of infection from one source in recent years. We also observed the rise in predicted Re values for HBV in Tuva, from less than 1 before 2000s to 5 after year 2000. Serosurvey of healthy volunteers have shown the average detection rate of HBsAg with anti-HDV to be 1.0% (95% CI: 0.57%–1.81%), which was significantly lower compared to data from 2008 serosurvey conducted in similar age cohorts of healthy volunteers (2.3% (26/1154, 95% CI: 1.53%–3.30%), p = 0.0218, Fisher exact test). No anti-HDV positive samples were detected in 2019 among participants under 30 years. HBsAg/anti-HDV positivity rate peaked at 7.4% in age group 50–59 years, which was significantly higher compared to similar age cohort surveyed in 2008 (1.6%, p < 0.0001).

**Conclusions: **Results of Bayesian analysis together with data from serosurvey have shown an increase in the intensity of the spread of HDV in Tuva in recent decades, which is apparently associated with HBV circulation in non-vaccinated adults and a large number of people living with HBV who are susceptible to HDV superinfection. Such silent HDV epidemics explains the high rates of liver cirrhosis and hepatocellular carcinoma observed in Tuva. This situation urges for implementation of HDV screening and prevention measures in the region, together with development of chemo- and immunotherapy aimed at HBV and HDV eradication.

**Acknowledgements: **This research was funded by a grant of the Russian Science Foundation (ID-20-15-00148).

### O9. MicroRNAs profiling in Senegalese HBV-infected patients

#### Rayana Maryse Toyé^1,2*^, Marie-Laure Plissonnier^1^, Gora Lô^2^, Maud Lemoine^3^, Yasmina Chouik ^1,4,5^, Souleymane Mboup^2^, Fabien Zoulim^1^, Massimo Levrero ^1,4,5^, Coumba Touré-Kane^2^ ** and Isabelle Chemin^1^**

##### ^1^Institut national de la santé et de la recherche médicale (Inserm) U1052, CRCL, Lyon, France; ^2^Institut de recherche en santé, de surveillance épidémiologique et de formation (Iressef), Diamniadio, Sénégal; ^3^Imperial College London, St Mary’s Hospital Campus, London, UK; ^4^Université Claude Bernard Lyon 1 (UCLB1), Lyon, France; ^5^Hospices Civils de Lyon (HCL), Lyon, France

###### Presenting author: Rayana Maryse Toye; ** Principal Investigators (isabelle.chemin@inserm.fr)

*Infectious Agents and Cancer* 2022, **17(Suppl 1)**: O9

**Background: **Among the 257 million hepatitis B virus (HBV) carriers worldwide, approximately 70 million live in Africa. The feared complications of the infection are cirrhosis and hepatocellular carcinoma (HCC). In Senegal, HBV is endemic and HCCs are often diagnosed at an advanced stage. Various factors appear to influence liver carcinogenesis, including viral genotype, exposure to aflatoxin B1 (AFB1) and regulation of microRNAs (miRNAs). MiRNAs are small noncoding RNAs that regulate gene expression and impact carcinogenesis. The aim of this work was to profile circulating miRNAs in chronic HBV carriers (CHB) and HCCs in Senegal.

**Materials and methods: **Serum samples were collected between 2013 and 2016 as part of the Prolifica project. Approximately 800 circulating miRNAs were profiled using NanoString's nCounter® technology in a retrospective cohort of 34 Senegalese patients (17 CHB and 17 HBV-related HCCs).

**Results: **A total of 404 miRNAs were detected. Principal component analysis (PCA) of the circulating miRNAs revealed that 58 miRNAs were quantified in at least 75% of patients. No differences were observed according to age, viral load, HBe status or clinical parameters such as ALT, AST. Nine miRNAs (let-7 g-5p, miR-122-5p, miR-181a-3p, miR-210-3p, miR-2682-5p, miR-300, miR-451a, miR-514a-3p and miR-519c-3p) were significantly differentially detected (p < 0.05) in the HBV-related HCCs as compared to CHB. Six of these miRNAs (let-7 g-5p, miR-122-5p, miR-210-3p, miR-300, miR-451a and miR-519c-3p) shared a network of regulatory pathways and target genes (e.g. MYC, BCL2L1, HIF1A, and BMI1) involved in the regulation of cell proliferation and immune system.

**Discussion and conclusions: **This study highlights the modification of the expression of several miRNAs in HBV-related HCC in Senegalese patients. In particular, miR-122-5p is strongly downregulated in HCCs and might represent a good candidate biomarker to be combined in a composite clinical diagnosis/prognostic score. The potential of these differentially detected circulating miRNAs for HCC prediction in CHB needs to be evaluated in a larger, longitudinal cohort.

### O10. Dynamics of LILRB2 immune-checkpoint in HIV and SIV infections

#### Benoit Favier

##### Center for Immunology of Viral, Auto-Immune, Hematological and Bacterial Diseases (IMVA-HB/IDMIT), Université Paris-Saclay, Inserm, CEA

##### Presenting author: Benoit Favier (benoit.favier@cea.fr)

*Infectious Agents and Cancer* 2022, **17(Suppl 1)**: O10

Dendritic cells (DCs) play an important role in initiating and regulating adaptive immune responses leading to the control of viral infections. However, previous reports have shown that HIV infection dysregulates DC functions that may account for viral persistence. Further studies indicate that this dysregulation is induced by the interaction of the Leukocyte immunoglobulin-like receptor subfamily B member 2 (LILRB2) inhibitory receptor with its MHC-I ligands and is associated with the rate of progression of the disease. In this context, we investigated the dynamics of the LILRB2/MHC-I inhibitory axis in DCs during the different phases of the infection. Our results show that patients in the primary phase of HIV infection show a strong increase of LILRB2 and MHC-I expression on the surface of DCs. We then used a macaque model of SIV infection to better characterize the dynamics of LILRB2 and MHC-I in the early phase of infection in blood and tissues. Our data show an up-regulation of LILRB2 and MHC-I on DCs but also macrophages, from one week after the onset of SIV infection. Overall, our results identify LILRB2 as a potential target to improve DC functions and thus anti-viral adaptive immune responses. We also discuss our latest tools to assess the role of LILRB2 in vivo and the potential of our strategy for the development of new immunotherapies against HIV.

### O11. The CD4 + CCR6 + T cell compartment is unique in EC compared to long-term ART treated individuals

#### Sara Svensson Akusjärvi^1*^, Shuba Krishnan^1^, Anoop T. Ambikan^1^, Soham Gupta^1^, Jimmy Esneider Rodrigues^2^, Ákos Végvári^2^, Maike Sperk^1^, Anders Sönnerborg^1,3^, Ujjwal Neogi^1,4,5^

##### ^1^Division of Clinical Microbiology, Department of Laboratory Medicine, Karolinska Institutet, ANA Futura, Campus Flemingsberg, 141 52 Stockholm, Sweden; ^2^Division of Chemistry I, Department of Medical Biochemistry and Biophysics, Karolinska Institutet, Campus Solna, 171 65 Stockholm, Sweden; ^3^Division of Infectious Disease, Department of Medicine Huddinge, Karolinska Institutet, I73, Karolinska University Hospital, 141 86 Stockholm, Sweden; ^4^Christopher S. Bond Life Sciences Centre, University of Missouri, Columbia, MO 65211, USA; ^5^Manipal Institute of Virology (MIV), Manipal Academy of Higher Education, Manipal, Karnataka, India

###### Presenting author: Sara Svensson Akusjärvi (sara.svensson.akusjarvi@ki.se)

*Infectious Agents and Cancer* 2022, **17(Suppl 1)**: O11

**Background: **Infection of HIV-1 induces a chronic inflammatory environment in the body that is not restored by antiretroviral therapy (ART). Although suppressive ART reduces infection to a minimal residual disease, the virus persists in different cell subsets in people living with HIV (PLWH) [1]. Persisting HIV-1 in combination with modulated immune activity and function of cell subsets may contribute to HIV-1 pathogenesis and immune dysfunction [2]. Some individuals who naturally control the infection in absence of ART (herein, PLWH_EC_) have lower levels of inflammatory markers. Even as studies have shown the immune reconstitution during a short-term ART, the effect of long-term therapy is still not known. In this project, we aimed to investigate the role of some key chemokine receptors in PLWH_EC_ compared to PLWH on long-term suppressive therapy (PLWH_ART_).

**Materials and methods: **The cohort consisted of PLWH_EC_ (*n* = *14*), PLWH_ART_, (*n* = *54*), and HIV-1 negative controls (*n* = *18*). Initial flow cytometry was performed looking at CCR2, CCR3, CCR5, and CCR6 on lymphocytic (CD4 and CD8) cell subsets. Statistical analysis was performed using Mann–Whitney U-test (*p* < *0.05*). Furthermore, CD4^+^CCR6^+^ and CD4^+^CCR6^−^ cell subsets were sorted from PLWH_EC_ (n = 4), PLWH_ART_ (n = 6), and HC (n = 3), and TMT proteomics performed. Proteins were searched against the Swiss Prot Human database in Proteome Discoverer v2.5 (false discovery rate 1%). Proteomic data were normalized using Normalyzer DE, missing data imputed by employing nearest neighbour averaging and variations removed using Combat in R. Differential expression analysis was performed using R/Biodonductor package Limma and gene set enrichment using piano. Benjamini–Hochberg adjusted p-value less than 0.2 was considered as significant.

**Results: **Flow cytometry analysis identified a significant decrease of CCR6 and CCR2 on CD4^+^ and CD8^+^ T cells in PLWH_EC_ compared to PLWH_ART_ (CD4; *p* < *0.0001* and *p* < *0.0001*, respectively. CD8; *p* < *0.0001* and *p* < *0.0001*, respectively) and HC (CD4; *p* < *0.0001* and *p* < *0.0001*, respectively. CD8; *p* < *0.0001* and *p* < *0.0001*, respectively). PLWH_ART_ also exhibited reduced CCR2 expression on CD8^+^ T cells compared to HC (*p* = *0.0292*). From the sorted cell populations, CD4^+^ T cells expressed an enrichment of OXPHOS (adj *p* < *0.05*) and interferon-α response (adj *p* < *0.05*) and decreased glycolysis (adj *p* < *0.05*) in PLWH_EC_ compared to PLWH_ART_. Furthermore, CD4^+^CCR6^+^ cells specifically expressed an enrichment of apoptosis (adj *p* < *0.05*) and p53 signalling (adj *p* < *0.05*) in PLWH_EC_ compared to PLWH_ART_ not seen in CD4^+^CCR6^−^ cells.

**Conclusions: **Flow cytometry analysis revealed a unique expression profile of CCR2 and CCR6 in PLWH_EC_ while PLWH_ART_ were more similar to HC on lymphocytic cell populations. The proteomic profile of CD4^+^ T cells in PLWH_EC_ exhibited reduced proteins involved in glycolysis and increased OXPHOS indicative of altered metabolic profile compared to PLWH_ART_. Furthermore, the enrichment of apoptosis and p53 signalling in CD4^+^CCR6^+^ cells from PLWH_EC_ indicates a higher susceptibility towards cell death mechanisms. CD4^+^CCR6^+^ T cells have been proposed as highly permissive to HIV-1 and major contributors to the viral reservoir [3]. Therefore, the reduced frequency and metabolic modulation in CD4^+^CCR6^+^ cells, together with enrichment of proteins involved in cell death, could potentially aid in facilitating natural control of HIV-1.

**Acknowledgements: **This study was funded by the Swedish Research Council and Karolinska Institutet Stiftelser och Fonder.

**References**

1. Cohn, L.B., N. Chomont, and S.G. Deeks, The Biology of the HIV-1 Latent Reservoir and Implications for Cure Strategies. Vol. 27. 2020: Cell Host & Microbe.

2. Zicari, S., et al., Immune Activation, Inflammation, and Non-AIDS Co-Morbidities in HIV-Infected Patients under Long-Term ART. Viruses, 2019. **11**(3).

3. Lee, A.Y.S. and H. Korner, CCR6/CCL20 chemokine axis in human immunodeficiency virus immunity and pathogenesis. J Gen Virol, 2017. **98**(3): p. 338–344.

### O12. Programmed cell death protein 1 (PD-1) and programmed cell death ligand 1 (PD-L1) expression profile in primary and metastatic cervical cancer tissue

#### Sona Chowdhury^1*^, Maxim Vonsky^2,3^, Maria Isaguliants^4,5 6^, Teresa M. Darragh^7^, Joel M. Palefsky^1^.

##### ^1^Division of Infectious Diseases, Department of Medicine, University of California, San Francisco, CA, USA; ^2^D.I. Mendeleev All-Russian Institute for Metrology, St. Petersburg, Russia; ^3^Almazov National Medical Research Center, St. Petersburg, Russia; ^4^Gamaleya National Research Center for Epidemiology and Microbiology, Moscow, Russia; ^5^Peoples Friendship University of Russia (RUDN University), Moscow, Russia; ^6^Riga Stradins University, Department of Pathology, Riga, Latvia; ^7^Department of Pathology, University of California, San Francisco, CA, USA

###### Presenting author: Sona Chowdhury (Sona.Chowdhury@ucsf.edu)

*Infectious Agents and Cancer* 2022, **17(Suppl 1)**: O12

**Background: **The programmed cell death protein 1 (PD-1) and programmed cell death ligand 1 (PD-L1) axis plays key roles in balancing protective immunity and immunopathology. Cancers exploit PD-L1/PD-1 inhibitory pathway to prevent host immune surveillance. There is limited knowledge regarding the expression profile and function of PD-L1/PD-1 axis in HPV-associated cervical cancer (CC). The aim of this study was to assess the expression of PD-L1 and PD-1 in epithelial cells and immune cell (IC) infiltrates of primary and metastatic squamous cervical carcinomas (SCC).

**Methods: **We examined 27 cervical tissues including 16 SCC, 1 SCC and high-grade squamous intraepithelial lesion (HSIL), 1 HSIL alone, 1 benign and 8 metastatic lymph nodes (LN), mainly from women living with HIV co-infected with tuberculosis. PD-L1 and PD-1 expression was quantified by immunohistochemistry (IHC) with anti-PD-L1 (Ventana SP263) and anti-PD-1 (Roche NAT105) antibodies.

**Results: **Of 17 primary SCC, PD-L1 was expressed in epithelial cells in 71% (n = 12), 29% (n = 5) were negative. PD-L1(+)-SCC samples revealed heterogeneous PD-L1 expression patterns in-between tumors and within each tumor. Membranous PD-L1 epithelial staining demonstrated distinct patterns: focal (66%), diffuse (8%), at the center (16.7%) or periphery (16.7%) of invading nests, and at the tumor-stroma interface (8%). LNs (n = 8) consisted of both tumor tissue and non-malignant residual lymph node tissue. 62% of metastatic LNs exhibited PD-L1 expression in epithelium with focal (37%), diffuse (12%) patterns, both in the center (12%) and at the tumor-stroma interface (12%). PD-L1 expression in epithelial cells was observed only in 1 HSIL sample. PD-L1 positive immune cells (ICs) were observed in the stroma of PD-L1(+)-SCC and were manually scored as: few (+ +), in 25%; moderate (+ + +), in 33%; and numerous (+ +  + +), in 16.7% of samples. Tumors negative for PD-L1 were all positive for PD-L1 expression in ICs in the stroma. In 50% of metastatic LN samples, PD-L1(+)-ICs were observed in the stroma irrespective of PD-L1 expression in tumor cells.

Epithelial cells did not express PD-1 in either SCC, or HSIL, or LN tissues. PD-1 expression was observed in stromal ICs irrespective of epithelial PD-L1 expression status. We semi-quantitatively scored the numbers of PD-1(+) ICs. 59% of primary SCC had moderate (+ + +) and 12% had numerous (+ +  + +) PD-1( +) ICs in the stroma. PD-1( +) ICs were observed in both HSIL samples. In the stroma of LNs, PD-1 expression in IC varied from negative, rare (+) in 12.5% samples, and few (+ +) in 50% of the samples irrespective of PD-L1 expression status in tumor cells. In the benign lymph node, PD-L1( +) and PD-1( +) ICs were clustered mostly in germinal centers of lymph nodes. The manual IHC scoring was further validated by a bioimage analysis software.

**Conclusion: **Varied PD-L1 expression patterns were observed both in primary and metastatic SCC. PD-L1 expression was seen not only in epithelial cells but also in ICs. In contrast, PD-1 expression was restricted to ICs. Level of PD-L1 expression varied in-between SCC samples and within one sample. This heterogenous pattern of PD-L1/PD-1 expression may contribute to immune-mediated pathogenesis of CC and warrants further investigation.

**Acknowledgements: **NIH5R01CA217715-03; RFBR17-54-30002.

### O13. Comparative gene expression and gene correlation analysis reveals different landscape of genes interaction in colon cancer

#### Ivan D. Trotsenko^1,2*^, Vladimir K. Bozhenko^3^, Uglesha S. Stanoevich^4^, Irina V. Stanoevich^5^, Egor N. Grebenkin^4^

##### ^1^Peoples Friendship University of Russia (RUDN University), Moscow, Russia; ^2^Moscow oncological society; ^3^Russian Scientific Center of Roentgenoradiology, Moscow, Russia; ^4^Kursk Oncological Research and Clinical Center named after G.E. Ostroverkhov, Kursk, Russia; ^5^Oncology department of Kursk State Medical University, Kursk, Russia

###### Presenting author: Ivan D. Trotsenko (trotsenkoivan@mail.ru)

*Infectious Agents and Cancer* 2022, **17(Suppl 1)**: O13

**Background: **The concept of a molecular phenotype, in particular for colon cancer (CC), implies the pooling of samples characterized by stable expression level of a set of genes in one group. Also high expression level is considered as evidence of gene functional activity in a phenotype. At the same time, the mode of gene interactions is not investigated taking into account their correlation coefficients.

**Materials and methods: **Using RT-PCR we compared expression levels and correlation coefficients of 43 genes, controlling proliferation and differentiation (*MYC, NDRG1, KI67, erbB2, GRB7, PTEN, CCNB1, ESR1, STK15 (AURKA), MYBL2, P16INK4A (CDKN2A), IGF1, IGF2, TGFb1, GREM1, LIF, LIFR), apoptosis (BIRC5, BCL2, BAG1, BAX, MMP9*), immune response and inflammation (*CD68, GNLY, COX-2 (PTGS2), IL2RA, IL12A, IL7, IL15, IL1B, IL10, CD45 (PTPRC), CD69, TLR2, TLR4, TPA (PLAT), HLA-G*), matrix remodeling and angiogenesis (*VEGFA* (subunits 165, 189), *MMP11, MMP2, MMP7, PAPPA, LGALS1*) in a cohort of 376 tissue samples. Cohort included: 77 normal colon (NC) samples taken from patients with benign colon adenomas, 132 adjacent mucosa (AM) and 167 samples of colon cancer (tumor—T), adjacent mucosa and tumor samples were taken from the same patient in pair. All samples were included into the analysis after morphological examination.

**Results: **We compared the result of tissue samples classification (T, AM and NC) on the basis of gene expression levels and gene correlation coefficients. Principal component analysis (PCA) of expression profile revealed significant activation of proliferative, martix metalloproteinases, antiapoptotic and angiogenic pathways activation in tumor compared to slight difference between NC and AM. On the PCA plot T samples were separated from NC and AM samples, the last two located in common field (Fig. 1A). These results show that the hallmark cancer pathways are activated in colon cancer as compared to adjacent colon mucosa and normal colon epithelium. Then we compared correlation matrices of gene expression for T, AM and NC samples by the same statistical analyses. According to PCA scores correlations of gene expression in NC differ significantly from correlations in T and AM samples (Fig. 1B) without great difference in last two groups. Cluster analysis revealed that correlation scores of genes in NC were significantly higher than correlation scores in T and AM (Fig. 1C).

**Fig. 1 (Abstract O13):**
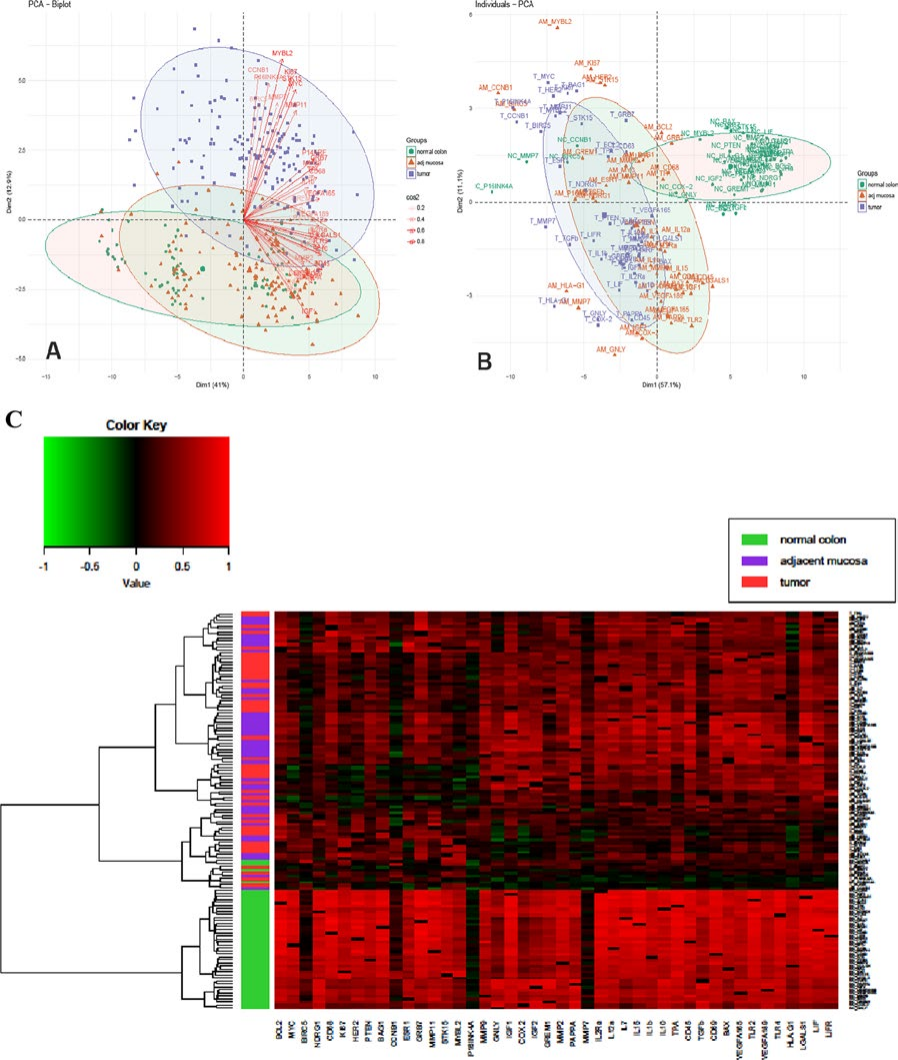
Gene expression and gene correlation profiles for NC, AM and CC. Principal component analysis for gene expression (**A**) and gene correlation (**B**) profiles; Heatmap of gene correlation profile (**C**)

**Discussion and conclusions: **Gene expression profile demonstrated a significantly higher level of proliferative activity, angiogenesis, apoptosis inhibitors, etc. in CC samples. At the same time, the interaction of these pathways in tumor and surrounding morphologically normal tissue is disrupted. This was demonstrated by comparison of gene correlation coefficients in tumor and normal epithelium. Despite the fact that the level of expression of cancer hallmarks in surrounding tumour mucosa and normal colon epithelium did not differ significantly, changes in intracellular pathways were significantly more coordinated in normal epithelium compared to tumor and adjacent mucosa, which was demonstrated by significantly higher correlation coefficients in NC. Thus, gene correlations analysis revealed violations of gene interaction not only in the tumor, but also in the surrounding morphologically unchanged epithelium.

## Session “immune response in chronic viral infections and cancer”

### PL8. Macrophages as viral targets and reservoirs for long-term survival

#### Julia Kzhyshkowska^1,2^

##### ^1^Laboratory of Translational Cellular and Molecular Biomedicine, Tomsk State University, Tomsk 634050, Russia; ^2^Institute of Transfusion Medicine and Immunology, Medical Faculty Mannheim, Heidelberg University, 68167 Heidelberg, Germany

###### Presenting author: Julia Kzhyshkowska (Julia.kzhyshkowska@medma.uni-heidelberg.de)

*Infectious Agents and Cancer* 2022, **17(Suppl 1)**: PL8

Viruses manipulate cell biology to utilize monocytes/macrophages as vessels for dissemination, long-term persistence within tissues and virus replication. Viruses enter cells through endocytosis, phagocytosis, macropinocytosis or membrane fusion. These processes play important roles in the mechanisms contributing to the pathogenesis of these agents and in establishing viral genome persistence and latency. Upon viral infection, monocytes respond with an elevated expression of proinflammatory signalling molecules and antiviral responses, as is shown in the case of the influenza, Chikungunya, human herpes and Zika viruses. Human immunodeficiency virus initiates acute inflammation on site during the early stages of infection but there is a shift of M1 to M2 at the later stages of infection. Cytomegalovirus creates a balance between pro- and anti-inflammatory processes by inducing a specific phenotype within the M1/M2 continuum. Despite facilitating inflammation, infected macrophages generally display abolished apoptosis and restricted cytopathic effect, which sustains the virus production. The majority of viruses employ monocytes/macrophages as a repository but certain viruses use these cells for productive replication. Viruses adapt their behavior to enter monocytes/macrophages, develop strategies for the immune escape, reprogramming of infected macrophages and manipulate their activity.

**References**

Nikitina E, Larionova I, Choinzonov E, Kzhyshkowska J. Monocytes and Macrophages as Viral Targets and Reservoirs. Int J Mol Sci. 2018 Sep 18;19(9):2821. https://doi.org/10.3390/ijms19092821.

### PL9. Immune-metabolic characterizations in HIV-infection—from elite controllers to patients on long-term successful therapy

#### Ujjwal Neogi^1,2^

##### ^1^The Systems Virology Lab, Division of Clinical Microbiology, Department of Laboratory Medicine, Karolinska Institute, ANA Futura, Campus Flemingsberg, Stockholm, Sweden; ^2^Manipal Institute of Virology (MIV), Manipal Academy of Higher Education, Manipal, Karnataka, India

###### Presenting author: Ujjwal Neogi (ujjwal.neogi@ki.se)

*Infectious Agents and Cancer* 2022, **17(Suppl 1)**: PL9

HIV-1 elite controllers (EC) are a rare but heterogeneous group of HIV-1-infected individuals who can suppress viral replication in the absence of antiretroviral therapy. The mechanisms of how EC achieve undetectable viral loads remain unclear. We aimed to investigate host plasma and faecal metabolomics and targeted plasma proteomics in a Swedish HIV-1 cohort including EC and treatment-naive viremic progressors (VP) as well as HIV-negative individuals (HC) to get insights into EC phenotype. Plasma metabolites belonging to antioxidant defense had higher levels in EC relative to VP, whereas inflammation markers were increased in VP compared with EC. Only four plasma proteins (CCL4, CCL7, CCL20, and NOS3) were increased in EC compared with HC, and CCL20/CCR6 axis can play an essential role in EC status [1]. We also investigated the fecal metabolome and microbiome. We observed an enrichment of dipeptides in EC compared to the other two study groups. The in silico and in vitro analyses identified anti-HIV-1 properties for two dipeptides that could bind to the HIV-1 gp120 and act as an HIV-1 antagonist. Furthermore, these dipeptides supported bacterial growth of the genus *Prevotella *in vitro that was enriched in EC, which influences host metabolism. Thus, increased levels of both dipeptides and *Prevotella* could provide beneficial effects for EC [2].

Given that EC is rare group of individuals, we next started looking people living with HIV (PLWH) with long-term treatment. Our study investigated alterations in the plasma metabolic profiles by comparing PLWH on long-term cART(> 5 years) and matched HIV-negative controls (HC) in two cohorts from low- and middle-income countries (LMIC), Cameroon, and India, respectively, to understand the system-level dysregulation in HIV-infection. Using untargeted and targeted LC–MS/MS-based metabolic profiling and applying advanced system biology methods, an altered amino acid metabolism, more specifically to glutaminolysis in PLWH than HC were reported. Another study combining both metabolomics and immune-phenotyping indicated altered glutamate metabolism associated with metabolic syndrome (MetS) in PLWH, which has clinical significance [3]. Further, modulation of cellular glutaminolysis promoted increased cell death and latency reversal in pre-monocytic HIV-1 latent cell model U1, which may be essential for the clearance of the inducible reservoir in HIV-integrated cells.

**References**

1. Sperk M, Mikaeloff F, Svensson-Akusjärvi S, Krishnan S, Sivasankaran M P, Ambikan AT, Nowak P, Sönnerborg A, Neogi U^†^ (2021) Distinct lipid profile, low-level inflammation and increased antioxidant defense signature in HIV-1 elite control status. *iScience (Cell Press)* 24:102111.

2. Sperk M, Ambikan AT, Ray S, Singh K, Mikaeloff F, Ceña Diez R, Narayanan A, Vesterbacka J, Nowak P, Sönnerborg A, Neogi U^†^ (2021) Novel mechanism of HIV elite control by enriching gut dipeptides as HIV-1 antagonist but *Prevotella* agonist. *J Virol J*ul 7:JVI0047921. https://doi.org/10.1128/JVI.00479-21.

3. Gelpi M, Mikaeloff F, Knudsen AD, Benfeitas R, Krishnan R, Svensson-Akusjärvi S, Høgh J, Murray DD, Ullum H, Neogi U, Nielsen SD (2021) Central role of the glutamate metabolism in long-term antiretroviral treated HIV-infected individuals with metabolic syndrome: a cross-sectional cohort study. *Aging (Albany NY)*. 2021 Oct 11;13. https://doi.org/10.18632/aging.203622.

### PL10. Metabolic dysfunction of natural killer cells in cancer; nutrients and metabolites play their part

#### David Finlay

##### Trinity Biomedical Sciences Institute, Trinity St. James’s Cancer Institute, Trinity College Dublin, Ireland

###### Presenting author: David Finlay (FINLAYD@tcd.ie)

*Infectious Agents and Cancer* 2022, **17(Suppl 1)**: PL10

Natural Killer (NK) cells are key immune cells that can identify and kill tumour cells. However, they are often found to be dysfunctional in cancer patients. Our work has explored the metabolic requirements of NK cell and how these relate to the anti-tumour effector functions of these cytotoxic lymphocytes. This work has led us to an appreciation that both an excess or deficit of nutrients within a tumour microenvironment might cause NK cell dysfunction. When considering how the metabolic conditions within the TME can impact upon NK cells it is important to examine the nutrient transporters expressed on NK cells, such as Slc7a5 and CD71, and the activity of various nutrient sensing signalling molecules including mTORC1, cMyc and Srebp. We propose supporting NK cell metabolism within the TME as a potential route towards improved of NK cell-based cancer immunotherapies.

### O14. Impairment of NK-DC cross-talk as a component of immune dysfunction in HIV-1 infection

#### Jorma Hinkula*, Anirban Sengupta, Cecilia Svanberg, Marie Larsson

##### BKV, MIIC, Linköping University, 58185 Linköping, Sweden

###### Presenting author: Jorma Hinkula (jorma.hinkula@liu.se)

*Infectious Agents and Cancer* 2022, **17(Suppl 1)**: O14

**Background: **Human immunodeficiency virus type 1 (HIV-1) is well known for its capacity to drive the immune system into inefficiency and immune suppression in infected individuals. The typical adaptive immune cells affected both direct and indirect are the CD4 + T helper cells and the cytotoxic CD8 + T cells driven into anergy and apoptosis. Less studied are the NK cells and the influence on their antiviral activities when exposed to HIV-1 by contacts with dendritic antigen-presenting cells (DC). NK cells have been presented as important antiviral activities in control of HIV infection, but seem to perform less well in individuals with progressive disease. The mechanisms for these phenomena are however poorly understood. We wanted therefore to collect and present some data that may shed light on how NK cells participate and loose or develop weaker antiviral activities due to cross-communication with antigen-presenting cells, such as DC. The aim of this study was to reveal the impact of HIV-1 in its free virion or complement or immune-complex forms as exposed on DC cell-surface for antigen-presentation and immune activation.

**Materials and methods: **Autologous DC and NK cell cultures, HIV-1 cultures, FACS analyses to analyze cell-markers, Cytokine secretion ELISAs, Statistical analyses using One-way Anova with Bonferroni post-test to analyze significant statistical differences.

**Results and discussion: **Immature dendritic cells exposed to free HIV-1 remain capable of supporting enhanced NK cell antiviral cell-signaling and activity. Instead, exposure to HIV-1 complexed with complement factors of immunoglobulin complexes prior to take up by DCs, seems to result in suboptimal and considerably more limited antiviral activity. One major condition to provide an immune reaction suppression in NK cells seems to be the cell-to-cell contact between the NK-and-DC cells exposed to the different HIV-1 presentation options. This lecture will discuss the different possible explanations on how and why these phenomena occur.

### O15. Impaired production of pro-inflammatory cytokines in HIV-infection associated with tuberculosis

#### Marina N. Nosik^1*^, Natalia V. Petrakova^2*^, Alexey V. Kravtchenko^3^, Uliana A. Kuimova^3^, Alexander Sobkin^4^

##### ^1^Mechnikov Institute of Vaccine and Sera, Moscow, Russia; ^2^Gamaleya Research Center for Epidemiology and Microbiology, Moscow, Russia; ^3^Central Research Institute of Epidemiology, Moscow, Russia; ^4^G.A. Zaharyan Moscow Tuberculosis Clinic, Moscow, Russia

###### Presenting authors: Marina N. Nosik (mnossik@yandex.ru), Natalia V. Petrakova (nvpetrakova@hotmail.com)

*Infectious Agents and Cancer* 2022, **17(Suppl 1)**: O15

**Background: **The symptoms of tuberculosis (TB) associated with HIV infection are often non-specific. The atypical course of TB in the late stages of HIV-infection leads to the late detection of M. tuberculosis infection/delayed diagnosis of TB, resulting in HIV-1/TB co-infected patients not receiving the TB treatment with prolonged circulation of M. tuberculosis. Circulation of *M. tuberculosis* in HIV-1 patients has profound effects on the immune system, it negatively regulates T-cell activation while activating HIV-1 replication. Specifically, co-infection dysregulates the production of cytokines necessary to control both infections, aggravating disease progression and compromising the effects of ART and TB treatment. Study aimed to characterize the expression of pro- and anti-inflammatory cytokines in patients with HIV- and TB monoinfections and HIV/TB co-infection before and during antiretroviral (ART)/anti-tuberculosis therapy (ATT), and to identify predictive markers of treatment efficacy, morbidity and mortality.

**Materials and methods: **A total of 351 individuals were enrolled: 111 patients with HIV/TB co-infection (22 patients were subsequently excluded from the study due to non-compliance with the treatment regimen), 80 patients with HIV monoinfection, 78 patients with TB monoinfection and 82 healthy controls. Plasmatic levels of IFN-γ, TNF-α, IL-6, IL-8, IL-17, Il-18 were detected by ELISA EIA-BEST Kit (Vector-Best, Moscow, RF) before initiation of therapy (both, ART and ATT) and then at 30, 60, 90 and 150–180 days after initiation of treatment. Cytokine concentrations were determined using calibration curve built using standards provided in each kit (sensitivity 0–5 pg/mL), and expressed in pg/mL. Statistics was done using IBM SPSS Statistics 17.0.

**Results: **We observed reduced secretion of IFN-γ and TNF-α in patients with HIV/TB co-infection compared to patients with HIV and TB mono-infections: IFN-γ—1.7- and 2.3 (p = 0.002); TNF-α—1.6- and 2.1- (p = 0.002), respectively. Also we found a 5.1-fold decrease in production of IL-17, which plays a key role in the formation of granulomas and the control of bacterial growth, in patients with HIV/TB co-infection, compared to patients with TB (p < 0.001) and 3.5-fold decrease compared to patients with HIV (p < 0.003). Correlation of IL-17 levels with the severity of TB forms was detected. The IL-17 secretion was reduced by almost 12 times in disseminated TB and 3.5 times in infiltrative TB (p = 0.001 and p = 0.002, respectively). On contrary, secretion of IL-6, IL-8 and IL-18 was significantly higher in HIV/TB patients compared to patients with HIV and TB mono-infections. IL-6: 9.8–fold increase vs HIV and 11.3-fold increase vs TB, p = 0.001; IL-8: twofold increase vs the group of patients with HIV mono-infection and 2.2-fold increase vs the TB group, p = 0.001. High levels of IL-6 and IL-8 persisted in patients with HIV/TB after 6 months of therapy. Multivariate regression analysis revealed an association between the level of IL-8 expression and treatment outcome, namely, an increase in the number of fatal outcomes in subgroups of HIV/TB-patients with high compared to low IL-8 levels (131.9 pg/ml (75 ÷ 145) vs 24.9 pg/ml (2 ÷ 65), p < 0.01, respectively; OR = 4.1 (95% CI 2.68–12.62, p = 0.003). Patients with co-infection with HIV/TB showed an increase in IL-18 expression compared to HIV-infected patients by 4.6 times; with TB patients by 3.3 times and with healthy donors by 22.4 times, respectively (p < 0.001). We also revealed a correlation between increased levels of IL-18 and the failure of therapy: on the 180 day of treatment the expression of IL-18 was by 5.6 times higher in patients with the failure of therapy comparing to other groups (p = 0.002).

**Discussion: **The decreased levels of proinflammatory cytokines in patients with dual infection indicate a prolonged dysregulation of the innate immune response and suggest ineffective immune reconstitution which aggravates disease severity in pulmonary/extrapulmonary TB in HIV/TB patients. IL-6 is a major factor in chronic inflammatory diseases. During X-ray examination, a visible decay in the lung tissue is detected only when necrotic masses are rejected through the bronchi. The absence of communication with the bronchus does not mean an absence of destruction. The latter could be detected through hypersecretion of IL-6. Indeed, high levels of IL-6 manifest TB-induced lung cavitation [1] and high severity grade in chest X-ray analysis [2]. With this, persistently elevated IL-6 expression on the background of ART may serve as early markers of *M. tuberculosis* infection. The other pro-inflammatory cytokine IL-8 activates leukocytes, specifically neutrophils, and causes their chemotaxis into the focus of inflammation. Hypersecretion of IL-8 accelerates this process and enhances local inflammation and tissue damage, contributing to the pathogenesis of HIV/TB-co-infection. Besides, elevated IL-8 levels manifest the development of immune reconstitution inflammatory syndrome (IRIS); IRIS on the background of TB associates with poor prognosis and mortality [3], confirmed in this study. Increased levels of IL-18 cytokine, especially concomitant with reduced production of IFN-γ and TNF-α, may contribute to increased viral replication and disease progression [4]. The revealed correlation between the increased level of IL-18 and therapy failure advance this cytokine as a potential marker of treatment response. Furthermore, high levels of IL-18 contribute to chronic low-grade inflammation, and are associated with an increased risk of cardiovascular complications [5], metabolic disorders (including insulin resistance) [6] and non-AIDS associated cancers (non-Hodgkin’s lymphoma, cancers of lung, liver, kidney, skin) [7,8]. The reduced secretion of TNF-α and IL-17 results in inability of the body to form granulomas thus promoting M. tuberculosis spread and development of generalized TB. The latter is confirmed by correlation of IL-17 levels with the severity of TB forms. Since IL-17 also acts as tumor suppressor during the process of tumorigenesis by enhancing NK cell and CTL cell activation, and recruiting neutrophil, NK cell, CD4 + and CD8 + T cell infiltration into tumor tissues [9], its reduced levels would favor the development of cancer lesions.

**Conclusions: **Our data advance hypersecretion of IL-6 in HIV patients with TB-infection, as well as hypersecretion of IL-6 and IL-8, with an aggravated course of HIV/TB co-infection with severe lung damage, analogous to their role in lung lesions in the acute stage of SARS-CoV-2 [10], and in morbidity and mortality from lung cancer [11]. Next we plan to investigate if IL-6/IL-8 hypersecretion in the settings of HIV, TB infections and HIV/TB co-infection can predict morbidity and mortality associated with lung and other firms of cancer.

**References**

1. Rambaran S, Naidoo K, Lewis L et al. Effect of Inflammatory Cytokines/Chemokines on Pulmonary Tuberculosis Culture Conversion and Disease Severity in HIV-Infected and -Uninfected Individuals From South Africa (2021) Front Immunol, 12, 1123.

2. Shete A, Bichare S, Pujari V et al. Elevated Levels of Galectin-9 but Not Osteopontin in HIV and Tuberculosis Infections Indicate Their Roles in Detecting MTB Infection in HIV Infected Individuals (2020) Front Microbiol, 17, 11, 1685.

3. Vinhaes CL, Araujo-Pereira M, Tibúrcio R et al. Systemic Inflammation Associated with Immune Reconstitution Inflammatory Syndrome in Persons Living with HIV (2021) Life, 11(1), 65.

4. Feria MG, Taborda NA, Hernandez JC, Rugeles MT. HIV replication is associated to inflammasomes activation, IL-1β, IL-18 and caspase-1 expression in GALT and peripheral blood (2018), PLoS One, 13(4):e0192845.

5. C. Mullis, T. H. Swartz. NLRP3 inflammasome signaling as a link between HIV-1 infection and atherosclerotic cardiovascular disease (2020) Front Cardiovasc Med, vol. 7, p. 95.

6. Mohan, J.; Ghazi, T.; Chuturgoon, A.A. A Critical Review of the Biochemical Mechanisms and Epigenetic Modifications in HIV- and Antiretroviral-Induced Metabolic Syndrome (2021) Int J Mol Sci, 22, 12020.

7. Alderuccio JP, Olszewski AJ, Evens AM, et al. HIV-associated Burkitt lymphoma: outcomes from a US-UK collaborative analysis (2021), Blood Adv, 27;5(14):2852–2862.

8. Baker KJ, Houston A, Brint E. IL-1 Family Members in Cancer; Two Sides to Every Story. Front Immunol, (2019), 10:1197.

9. Nalbant A. IL-17, IL-21, and IL-22 Cytokines of T Helper 17 Cells in Cancer. J Interferon Cytokine Res, (2019), 39(1):56–60.

10. Giuseppe M. SARS-CoV-2 and COVID-19: Is interleukin-6 (IL-6) the ‘culprit lesion’ of ARDS onset? What is there besides Tocilizumab? SGP130Fc (2020) Cytokine: X, 2(2).

11. Ryan BM, Pine SR, Chaturvedi AK et al. A combined prognostic serum interleukin-8 and interleukin-6 classifier for stage 1 lung cancer in the prostate, lung, colorectal, and ovarian cancer screening trial (2014) J Thorac Oncol, 9(10), 1494–14503.

### O16. Identification by peptide microarray analysis of anti-HCV specific antibody response in patients with HCV-related chronic liver diseases, hepatocellular carcinoma and lymphoproliferative disorders

#### Anna Lucia Tornesello^1*^, Ulf Reimer^2^, Pavlo Holenya^2^, Tobias Knaute^2^, Francesco Izzo^3^, Luigi Buonaguro^1^, Maria Lina Tornesello^1^ and Franco Maria Buonaguro^1^

##### ^1^Molecular Biology and Viral Oncology Unit, Istituto Nazionale Tumori – IRCCS Fondazione Pascale, Napoli, Italy; ^2^JPT Peptide Technologies GmbH – Volmerstrasse 5 - 12489 Berlin, Germany; ^3^Hepatobiliary Surgery, Istituto Nazionale Tumori – IRCCS Fondazione Pascale, Napoli, Italy

###### Presenting author: Anna Lucia Tornesello (a.tornesello@istitutotumori.na.it)

*Infectious Agents and Cancer* 2022, **17(Suppl 1)**: O16

**Background: **Hepatitis C virus (HCV) infection is a progressive disease that may result in chronic active hepatitis, cirrhosis, hepatocellular carcinoma and lymphoproliferative disorders. It is estimated that about 160 million individuals, (2.35% of the world population) are chronically infected with HCV. HCV induces chronic hepatitis in more than 90% of infected subjects which may evolve in liver cirrhosis (30% of cases) and finally in hepatocellular carcinoma (HCC) (3%). Current progression markers are mainly based on biochemical evaluation of liver damage (elevation of alanine and aspartate transaminases) and inflammation (elevation of alpha-fetoprotein). Such markers are not specific for HCV-related liver disease and are elevated also for other infections (i.e. HBV and HCMV) or metabolic disorders (i.e. steatosis). Predictive biomarkers are helpful in matching HCV co-factors and to select high priority people for direct anti-viral treatment. The main objective of this study was the identification of new biomarkers specific for HCV-related neoplasia among chronically HCV infected subjects by HCV-based peptide array analysis.

**Materials and methods: **To perform a cohomprensive epitope mapping of anti-HCV antibodies, we analized 71 plasma samples of positive subjects, including 49 HCC patients, nine cryoglobulinaemic patients, and 13 asymptomatic HCV chronic infected subjects on a peptide microarray platform made of 5952 overlapping 15-mer synthetic peptides covering the whole HCV proteome (C, E1, E2, NS2, NS3, NS4A, NS4B, NS5A, NS5B and P7).

**Results and discussion: **Overall, the level of anti-HCV antibodies was significantly higher in HCV-positive cryoglobulinaemic subjects versus all other groups of HCV-positive patients. Particularly, a statistically significant stronger immune response was observed against the C-terminal peptides of the C protein in cryoglobulinaemia *versus* HCV chronic infected subjects (*P* < 1*10^–6^). Both HCC and MC showed a significant stronger immune response against peptides of C, E2, NS3, NS4A, NS4B, NS5A and p7 compared to HCV positive subjects with chronic hepatitis.

### O17. Enzymatic activities of RNA dependent RNA polymerase in chronic hepatitis C and their clinical correlates

#### Juris Jansons^1,2^, Tatiana Tallo^3^, Elena Royo Rubio^1,2,4^, Alexey G. Prilipov^5^, Alexander V. Ivanov^5,6^, Stefan Petkov^7^, Ingrida Mitre^1^, Elizaveta Starodubova^6^, Olga V. Masalova^5^, Olga O. Znoiko^8^, Olga V. Kalinina^3,9^, Jin-Ching Lee^10^, Maria G. Isaguliants* ^1,5,7,11^

##### ^1^Riga Stradins University, Riga, Latvia; ^2^Latvian Biomedical Research and Study Center, Riga, Latvia; ^3^Public Health Agency of Sweden, Stockholm, Sweden; ^4^Laboratorio Inmunobiología Molecular, Instituto de Investigación Sanitaria Gregorio Marañón, Madrin, Spain; ^5^Gamaleya National Research Center for Epidemiology and Microbiology, Moscow, Russia; ^6^Engelhardt Institute of Molecular Biology, Moscow, Russia; ^7^Department of Microbiology, Tumor and Cell Biology, Karolinska Institutet, Stockholm, Sweden; ^8^Moscow State University of Medicine and Dentistry named after Evdokimov, Moscow, Russia; ^9^Federal Almazov North-West Medical Research Centre, St Petersburg, Russia; ^10^Department of Biotechnology, Kaohsiung Medical University, Taiwan; ^11^Peoples Friendship University of Russia (RUDN University), Moscow, Russia

###### Presenting author: Maria G. Isaguliants (maria.issagouliantis@rsu.lv)

*Infectious Agents and Cancer* 2022, **17(Suppl 1)**: O17

**Background: **In chronic hepatitis C (CHC), liver enzyme levels correlate with histological parameters, and high levels are associated with liver damage. Contradictory data exists on the dependence of liver damage on HCV RNA levels, from high viral load correlating with liver enzyme levels and liver injury [1, 2] to inverse correlation of viral load with ALT but positive association of the latter with histological severity of liver disease [3]. A key determinants of HCV replication competence is the enzymatic activity of RNA-dependent RNA-polymerase (RdRp). Still, relation of RdRp activity to liver disease is largely unknown except for one observation of direct association of RdRp activity with ALT levels [4]. The aim of this study was to define the relations between RdRp activities and clinical course of CHC including HCV serum load, liver enzyme levels and/or liver injury.

**Materials and methods: **HCV RNA of 17 CHC patients (CHCP) with persistently normal (PNAT) and 25 patients with abnormal ALT levels on ≥ 3 occasions (ABN) was reverse transcribed and amplified, and RdRp-encoding region was sequenced (ABI3130xl). Gene sequences were assembled using DNAStar software (Lasergene) and deposited in GeneBank. cDNAs were cloned into pGEM-T, clones were sequenced. RdRp sequence alignments were created using MUSCLE, consensus sequences were generated with Geneious 8.1.2. RdRp covariance network was created as described [5] and visualized in Cytoscape 3.1.1. Eight RdRp variants from ABN and 9 from PNAT patients identical to those represented by cDNA were recloned into pVax1 for eukaryotic expression, generating 17 pVaxNS5B expression vectors. Expression in HEK cells was confirmed by PAGE with Western blotting using rabbit anti-NS5B. Specific RdRp activity in eukaryotic cells was evaluated using a cell-based reporter assay employing a plasmid containing firefly luciferase (Luc) gene regulated by host polymerase and Renilla luciferase gene in reverse orientation flanked by negative strands of the HCV 5'- and 3'-UTRs and regulated by HCV RdRp [6]. Capacity of RdRp to induce expression from IFN-b promoter was assessed by co-transfection of HEK cells with pGL3-IFNb (Addgene) and each of 17 pVaxNS5B variants, luminescence was recalculated per number of viable cells, and as fold increase to controls. Empty vector and pVax expressing enzymatically active and inactivated HIV-1 reverse transcriptase (RT_A) were used as controls [7]. Measurements were done in three replicas, each in duplicate. Statistical analysis was done using STATISTICA 11.0 (TIBCO).

**Results: **RdRps of ABN and PNAT patients revealed identical consensus sequences, the same covariance networks. ABN and PNAT RdRps did not differ in specific RdRp activity on HCV IRES. Specific activity was positively correlated with HCV viral load (R = 0.58; p = 0.02; Spearman test), and level of fibrosis (R = 0.85; p = 0.0001), but not with the liver enzyme levels or other parameters of Knodell Score. Capacity of RdRps to initiate RNA synthesis was measured indirectly by assessing Luc expression from IFN-b promoter, activated by dsRNA through RIG-1-regulated pathway. Both RdRps from ABN and PNAT CHCP suppressed IFN-b promoter-regulated Luc expression in vitro, the 1st by 20%, and 2nd—30–50% compared to cells transfected with reporter alone, as the enzymatically active, but not the inactivated HIV RT_A did. Effects on INF-b promoter of RdRps derived from PNAT and from ABN CHCPs did not differ, but correlated with the specific enzymatic activity of RdRps.

**Discussion and conclusions: **RdRps of CHC with ABN and PNAT had similar specific activity, which was not correlated to liver enzyme levels observed in the patients donating respective RdRp sequences. At the same time, specific activity correlated with viremia and liver fibrosis. Importantly, specific RdRp activity correlated with the capacity of RdRps to regulate the expression from type I interferon (IFN-b) promoter, known to occur via activation of RIG-I, indicating its role in the infammatory processes occurring in the liver, eventually leading to fibrosis. This demonstrates that CHCP with normal ALT levels should be followed-up as patients with elevated ALT levels because they can harbor actively replicating virus and suffer from pronounced liver disease.

**Acknowledgments: **Program NAWA EUROPARTNER: Strengthening and spreading international partnership activities of the Faculty of Biology and Environmental Protection for interdisciplinary research and innovation of the University of Lodz to MI at RSU, LZP-2020/2-0376 to JJ and MI, and RFBR 19-04-01034 to MI.

**References**

1. Adinolfi LE et al. Hepatology. 2001 Jun;33(6):1358–64. https://doi.org/10.1053/jhep.2001.24432.

2. Zechini B, Pasquazzi C, Aceti A. Eur J Gastroenterol Hepatol. 2004 Sep;16(9):891–6. https://doi.org/10.1097/00042737-200409000-00013

3. Yeo AE et al. J Viral Hepat. 2001 Jul;8(4):256–63. https://doi.org/10.1046/j.1365-2893.2001.00302.x.

4. Cao F et al. J Viral Hepat. 2011 May;18(5):349–57. https://doi.org/10.1111/j.1365-2893.2010.01316.x.

5. Donlin MJ et al. J Virol. 2012 Mar;86(6):3050–63. https://doi.org/10.1128/JVI.06857-11. Epub 2012 Jan 11.

6. Lee JC et al. Anal Biochem. 2010 Aug;403(1–2):52–62. https://doi.org/10.1016/j.ab.2010.04.004.

7. Jansons J et al. Microorganisms. 2021 May 17;9(5):1073. https://doi.org/10.3390/microorganisms9051073

### O18. Inflammation is reprogramming metabolic state of the organism via accumulation of IL-6

#### Maxim A. Nosenko^1,2*^, Anastasia S Yakovleva^1,3^, Denis E. Anisov^1,3^, Marina S. Drutskaya^1^, Sergei A. Nedospasov^1,3,4^

##### ^1^Engelhardt Institute of Molecular Biology, Russian Academy of Sciences, Moscow, Russia; ^2^Current affiliation: Trinity Biomedical Sciences Institute, Trinity College Dublin, Dublin, Ireland; ^3^Biological Faculty, Lomonosov Moscow State University, Moscow, Russia; ^4^Sirius University of Science and Technology, Federal Territory Sirius, Russia

###### Presenting author: Maxim A. Nosenko (maxim.nosenko@tcd.ie)

*Infectious Agents and Cancer* 2022, **17(Suppl 1)**: IL-6

Inflammatory conditions are accompanied by profound adaptations of both cellular and systemic metabolism. However, despite extensive investigation of the immune cell metabolism, less is known about the mechanisms of physiological metabolic shifts in the context of inflammation.

We employed mouse model of LPS-induced systemic inflammation that results in drastic reprogramming of metabolic state of the organism and demonstrated the key role of IL-6 in regulation of this adaptation. Genetic or pharmacological inactivation of IL-6 significantly abrogated inflammatory effect on glucose homeostasis. In particularly, we have shown that IL-6 regulates gene expression of key enzymes in the glycogen metabolism pathway in the liver of mice during inflammation. This resulted in significant hypoglycaemia and hypothermia in response to inflammatory conditions, that were not evident if IL-6 was inactivated. The involvement of glycogen metabolism pathway in regulation of glucose homeostasis during inflammation was further confirmed by pharmacological inhibition of glycogen phosphorylase. Finally, we found that IL-6 knock-out mice failed to induce fatigue upon LPS challenge as evidenced by RotaRod training. Fatigue, along with other well-described symptoms of so called “sickness behaviour”, could potentially contribute to the energy balance shift towards immune response rather than physical activity. We thus believe that via regulation of systemic metabolism IL-6 launches crucial physiological adaptations to the inflammatory conditions. Altogether our data demonstrate specific role of IL-6 in regulation of systemic metabolism and physiology in the context of inflammation. Further investigation of the mechanisms of these adaptations could potentially lead to the development of new therapies for infection- and cancer-induced multi-organ pathologies such as cachexia.

**Acknowledgements: **The work was supported by Russian Science Foundation grant #19-75-30032.

### O19. Itaconate and dimethyl itaconate promote LPS-induced expression of IL-6 in white adipose tissue

#### Denis E. Anisov*^1,2^, Anastasia S. Yakovleva^1,2^, Marina S. Drutskaya^1,3^, Sergei A. Nedospasov^1,2,3,4^, Maxim A. Nosenko^5^

##### ^1^Engelhardt Institute of Molecular Biology, Russian Academy of Sciences, Moscow, 119991, Russia; ^2^Lomonosov Moscow State University, Moscow, 119991, Russia; ^3^Center for Precision Genome Editing and Genetic Technologies for Biomedicine, Engelhardt Institute of Molecular Biology, Russian Academy of Sciences, Moscow, 119991, Russia; ^4^Sirius University of Science and Technology, Sirius, 354340, Russia; ^5^Trinity Biomedical Sciences Institute, Trinity College Dublin, Dublin 2, Ireland

###### Presenting author: Denis E. Anisov (denis.anisoff@gmail.com33333)

*Infectious Agents and Cancer* 2022, **17(Suppl 1)**: O19

**Background: **Specific metabolites play a profound immunoregulatory role under inflammatory conditions [1]. One of such metabolites is itaconate that was shown to regulate cytokine production. In particular, IL-6 secretion by macrophages in response to LPS is decreased in the presence of itaconate [2]. However, most of the effects of itaconate and its derivatives were extensively studied in macrophage cell cultures in vitro, whereas their functions in vivo remain poorly understood. In the current study, we investigated the effects of itaconate and its derivative dimethyl itaconate (DI) on cytokine production during inflammation in vivo*.*

**Materials and methods: **In vitro* effect of itaconate.* Murine bone marrow-derived macrophages (BMDM) were pretreated for 2 h with 0.25 mM of DI, followed by administration of 100 ng/mL of *E.coli* LPS. After 4 h of activation TNF and IL-6 accumulation were measured on protein and mRNA levels using ELISA and qPCR, respectively.

In vivo* effect of itaconate.* Mice were injected with 5 mg of ITA or DI, followed by 50 ug of LPS (non-lethal dose). 4 h after LPS injection blood and key metabolic organs were collected. Cytokine production was measured using ELISA and Multiplex Assay.

Experimental groups were compared using one-way analysis of variance (ANOVA) followed by Dunnett’s post-hoc test. The cytokine multiplex analysis data were processed using Principal Component Analysis (PCA).

**Results: **DI treatment restricts TNF and IL-6 production in LPS-activated BMDMs. However, both itaconate and DI led to increased IL-6 levels in the blood of LPS-treated mice, demonstrating the opposite effects of itaconate and its derivatives in vitro vs in vivo. In addition, administration of both itaconate and DI resulted in increased production of IL-10 and CXCL2, as well as in suppressed IFNγ accumulation in the blood of LPS-treated mice. Furthermore, we found that under the inflammatory conditions itaconate- and DI-dependent increase in *Il6* gene expression specifically occurred in white adipose tissue.

Altogether, we show that the effects of itaconate and its derivative, dimethyl itaconate, on inflammatory response may differ in vitro and in vivo. While the role of itaconate in suppressing IL-6 production was clearly demonstrated using BMDM cultures, we observed an opposite impact of itaconate and dimethyl itaconate on IL-6 expression in the mouse model of inflammation. We conclude that physiological functions of itaconate as well as potential therapeutic implications of its derivatives warrant further investigation.

**Conclusions**

1. DI restricts TNF and IL-6 production in LPS-activated bone marrow–derived macrophages;

2. DI and ITA contribute to increased accumulation of IL-6, IL-10, CXCL2 and to decreased accumulation of IFNγ in the blood of LPS-treated mice;

3. DI and ITA contribute to increased *Il6* expression in white adipose tissue of LPS-treated mice.

**Acknowledgements: **This work was supported by RSF grant № 19‐75‐30032.

**References**

1. Zasłona Z., O’Neill L.A.J. Cytokine-like Roles for Metabolites in Immunity. *Mol. Cell.* 78, 814–823 (2020).

2. Bambouskova, M. et al. Electrophilic properties of itaconate and derivatives regulate the IκBζ-ATF3 inflammatory axis. *Nature*. 556, 501–504 (2018).

3. ATF3 inflammatory axis. *Nature*. 556, 501–504 (2018).

## Session “approaches to viral infection and cancer cure”

### PL11. Virological cure of chronic hepatitis B virus infection: from «Is it possible?» to «When?»

#### Vladimir Chulanov^1,2,3^ and Dmitry Kostyushev^1,2^

##### ^1^National Medical Research Center of Tuberculosis and Infectious Diseases, Ministry of Health, 127994, Moscow, Russia; ^2^Scientific Center for Genetics and Life Sciences, Division of Biotechnology, Sirius University of Science and Technology, 354340 Sochi, Russia; ^3^Sechenov University, 119991 Moscow, Russia

##### Presenting author: Dmitry Kostyushev (dkostushev@gmail.com)

*Infectious Agents and Cancer* 2022, **17(Suppl 1)**: PL11

Chronic hepatitis B virus infection affects millions of people worldwide. Current treatment options can help to control viral infection and substantially reduce the risks of cirrhosis and hepatocellular carcinoma, but cannot eliminate the virus from infected cells and rarely achieve the so-called functional cure. A plethora of highly effective antiviral regimes and approaches is currently under development, including those targeting HBV transcription (siRNAs, SMC5/6, HBx, selective inhibitors of histone acetyltransferases etc.), cccDNA (site-specific nuclease, cccDNA destabilizers), HBsAg release, HBcAg proteins, immune modulation (agonists of Toll-like receptors, anti-PD1 antibodies, cytokine therapies, CAR T cells etc.), and approaches based on novel therapeutic vaccines. In this report, we provide the state-of-art in developing novel therapeutic approaches and identifying new therapeutic targets, assess their prospects to manage HBV infection and achieve the cure.

**Acknowledgements: **This work was funded by RSF grant 20-15-00373.

### PL12. The type of immune response is a critical factor in the immune battle against cancer

#### Alexandre Corthay^1,2^

##### ^1^Tumor Immunology Lab, Department of Pathology, Rikshospitalet, Oslo University Hospital, Oslo, Norway; ^2^Hybrid Technology Hub – Centre of Excellence, Institute of Basic Medical Sciences, University of Oslo, Oslo, Norway

###### Presenting author: Alexandre Corthay (a.b.corthay@ous-research.no)

*Infectious Agents and Cancer* 2022, **17(Suppl 1)**: PL12

The immune system has developed three main types of immune responses (type 1, 2, and 3) to optimally respond to different categories of pathogens and cancer. Each type of immune response is driven by a dedicated T helper (Th) cell subset. Th1 cells govern type 1 immunity that is protective against intracellular microbes and is also considered to be the most appropriate response to fight cancer. Th2 cells orchestrate type 2 immunity against parasites and venoms. Th17 cells control type 3 immunity to eliminate extracellular bacteria and fungi. T follicular helper (Tfh) cells participate in both type 1 and type 2 immune responses, while T regulatory (Treg) cells prevent immunopathology by suppressing detrimental immune responses of any type. The Th composition of human solid tumors remains poorly characterized. Therefore, we have recently established a four-color multiplex chromogenic immunohistochemical assay for detection of Th1, Th2, Th17, Tfh and Treg cells in human tumor sections. Using this new method, we found that human primary lung tumors are Th2-skewed and contain numerous Treg cells. If human tumors are Th2-skewed, as our data in lung cancer suggest, reprogramming the type of immune response from a detrimental Th2 to a beneficial Th1 may be critical to increase the response rate of immunotherapy for cancer.

### PL13. How to make cancer vaccines more effective? Importance of adjuvant strategy

#### Joon Haeng Rhee*

##### Combinatorial Tumor Immunotherapy MRC, Department of Microbiology and Clinical Vaccine R&D Center, Chonnam National University Medical School, South Korea

###### Presenting author: Joon Haeng Rhee (jhrhee@chonnam.ac.kr)

*Infectious Agents and Cancer* 2022, **17(Suppl 1)**: PL13

Cancer vaccines are designed to enhance tumor-specific cytotoxic T lymphocyte (CTL) responses resulting in tumor eradication or progression retardation. Despite sound scientific basis for therapeutic cancer vaccines, extensive efforts have failed to report successes through clinical applications. Compared with microbial antigens, cancer antigens derived from normal human cells are relatively less immunogenic. For successful cancer vaccines, we should find the ways how to potentiate host immune responses against less immunogenic cancer antigens. The components of cancer vaccines, the antigen, the delivery system, and the adjuvant, can have a significant impact on vaccine immunogenicity. As for cancer antigens, extensively tried tumor associated antigens were unsuccessful in inducing sufficient tumor suppression. Next-generation sequencing and improved bioinformatics tools have enabled better identification of tumor specific neoantigens, which are more desirable immunogens because they result from somatic mutations of normal genes and are more immunogenic than tumor associated antigens. However, even cancer specific neoantigens are still significantly less immunogenic than microbial antigens, which requires incorporation of potent adjuvants and strategic delivery systems. In this presentation, the importance of adjuvant in cancer vaccination will be discussed.

### O20. Rebound of HBV replication followed by transient CRISPR/Cas9 RNPs and its prevention by rcDNA depletion

#### Anastasiya Kostyusheva^1*^, Sergey Brezgin^1,2^, Natalia Ponomareva^1^, Ekaterina Bayurova^3,4^, Irina Goptar^5^, Anastasiya Nikiforova^5^, Anna Sudina^6^, Ilya Gordeychuk^3,4^, Alexander Ivanov^7^, Dmitry Kostyushev^1,2^ and Vladimir Chulanov^1,2,8^

##### ^1^National Medical Research Center of Tuberculosis and Infectious Diseases, Ministry of Health, 127994, Moscow, Russia; ^2^Scientific Center for Genetics and Life Sciences, Division of Biotechnology, Sirius University of Science and Technology, 354340 Sochi, Russia; ^3^Chumakov Federal Scientific Center for Research and Development of Immune-and-Biological Products of Russian Academy of Sciences, 108819 Moscow, Russia; ^4^National Research Center for Epidemiology and Microbiology named after NF Gamaleya, 123098 Moscow, Russia; ^5^Izmerov Research Institute of Occupational Health, Moscow, 105275, Russia; ^6^Federal State Budgetary Institution Centre for Strategic Planning and Management of Biomedical Health Risks of the Federal Medical Biological Agency, 119435, Moscow, Russia; ^7^Engelhardt Institute of Molecular Biology, Russian Academy of Sciences, Vavilov Str. 32, Moscow 119991, Russia; ^8^Sechenov University, 119991 Moscow, Russia

###### Presenting author: Anastasiya Kostyusheva (kostyusheva_ap@mail.ru)

*Infectious Agents and Cancer* 2022, **17(Suppl 1)**: O20

**Background: **Chronic hepatitis B virus (HBV) infection was reported in > 250 million people worldwide, causing over 1 million death annually. Developing novel therapeutics aimed at complete cure of the disease is urgently needed. CRISPR/Cas9 systems can effectively deplete the major form of the HBV genome, covalently closed circular DNA (cccDNA) and, potentially, disrupt viral replication and clear the virus from infected cells. The aim of this study was to test the effects of transient CRISPR/Cas9 delivery on HBV replication and develop CRISPR/Cas9-based strategies to clear the virus from cells.

**Materials and methods: **Streptococcus thermophilus StCas9 protein (CRISPR locus 1) (StCas9) protein was generated and complexed with in vitro transcribed highly potent sgRNA targeting conserved regions of the HBV genome (described previously). StCas9/sgRNAs were transfected to HepG2 cells together with rcccDNA using CRISPRMAX reagent (ThermoFisherScientific) or Lonza nucleofection system (Lonza). Intracellular delivery of StCas9 was analyzed by immunostaining with specific antibodies. In vitro cleavage activity of StCas9 with HBV-specific sgRNAs was tested at recombinant cccDNA (rcccDNA). Anti-HBV activity was measured by HBV transcription, total intracellular HBV DNA levels, rcccDNA levels, HBsAg measurement and anti-HBcAg immunostaining. On-target nucleolytic activity of Cas9 protein at HBV rcccDNA was assayed by NGS. All initial measurements were performed 3 days post CRISPR/Cas9 delivery. HBV rebound was analyzed by measuring HBV replication intermediates from day 4 to day 14. Alternatively, HepG2 cells were treated with HBV reverse transcription inhibitor lamivudine for 6 days followed by withdrawal and analysis of HBV markers for the next 14 days.

**Results: **Although CRISPR/Cas9 RNPs cleared over 99% of HBV intermediates by the 3rd day post intracellular delivery, HBV rebounded recovering replication by the 14th day. Treating HepG2 cells 1 day before CRISPR/Cas9 RNPs delivery and for 5 days post-delivery, prevented rebound of HBV replication.

**Conclusions: **Destroying > 99% of HBV rcccDNA is not sufficient for clearing HBV from cells. HBV rcDNA may form cccDNA de novo and result in viral rebound. Depleting HBV rcDNA or blocking rcDNA → cccDNA conversion step is necessary to eliminate replicating virus.

**Acknowledgements: **This work was funded by RSF grant 20-15-00373.

### O21. Antiviral activity of APOBEC3A and APOBEC3B induced by dSaCas9-p300 ribonucleoprotien complexes

#### Sergey Brezgin^1,2*^, Anastasiya Kostyusheva^1^, Natalia Ponomareva^1^, Ekaterina Bayurova^3,4^, Irina Goptar^5^, Anastasiya Nikiforova^5^, Anna Sudina^6^, Yurii Babin^1,**^, Ilya Gordeychuk^3,4^, Vladimir Chulanov^1,2,7^ and Dmitry Kostyushev^1,2^

##### ^1^National Medical Research Center of Tuberculosis and Infectious Diseases, Ministry of Health, 127994, Moscow, Russia; ^2^Scientific Center for Genetics and Life Sciences, Division of Biotechnology, Sirius University of Science and Technology, 354340 Sochi, Russia; ^3^Chumakov Federal Scientific Center for Research and Development of Immune-and-Biological Products of Russian Academy of Sciences, 108819 Moscow, Russia; ^4^National Research Center for Epidemiology and Microbiology named after NF Gamaleya, 123098 Moscow, Russia; ^5^Izmerov Research Institute of Occupational Health, Moscow, 105275, Russia; ^6^Federal State Budgetary Institution Centre for Strategic Planning and Management of Biomedical Health Risks of the Federal Medical Biological Agency, 119435, Moscow, Russia; ^7^Department of Infectious Diseases, Sechenov University, 119991 Moscow, Russia.

###### Presenting author: Sergey Brezgin (seegez@mail.ru); **Current address: MeLSyTech, Ltd, Dzerzhinsk, Russia

*Infectious Agents and Cancer* 2022, **17(Suppl 1)**: O21

**Background: **APOBEC (Apolipoprotein B mRNA Editing Catalytic Polypeptide-like) family of proteins play important roles in the defence of human cells to invading viruses [1]. Potent antiviral activity of APOBEC proteins is reported for different viral infections, including human immunodeficiency virus, herpes simplex virus, papilloma virus, hepatitis B virus (HBV) etc. [2]. High doses of IFN-α and agonists of TNF-α are able to induce expression of certain APOBEC factors and suppress viral replication. However, the use of these factors is limited due to toxicity and adverse effects. In turn, CRISPR/Cas systems fused to transcriptional activators enable direct activation of target gene expression [3]. The aim of this study was to develop a method for transient activation of APOBEC3A and APOBEC3B factors and test its anti-viral activity at HBV in vitro models.

**Materials and methods: **Nucleolytically-dead, high-fidelity variant of Staphylococcus aureus Cas9 protein fused to p300 acetyltransferase core subunit (dSaCas9-p300) recombinant protein was produced and purified. Single-guide RNAs (sgRNAs) targeting promoter regions of APOBEC3A (A3A) and APOBEC3B (A3B) genes were designed in ChopChop and UCSC Genome Browser. PCR-products encoding sgRNAs under T7 promoter were generated using overlap extension PCR, as described previously. sgRNAs were produced from PCR products by in vitro transcription using T7 polymerase. HepG2 cells were transfected with ribonucleoprotein complexes (RNPs) of dSaCas9-p300/sgRNAs using CRISPRMAX (ThermoFisherScientific) as such or together with recombinant covalently closed circular DNA (rcccDNA) of HBV. mRNA of target genes was measured with specific sets of primers and probes. HBV transcription, total intracellular DNA and rcccDNA levels were measured by PCR by appropriate protocols with specific sets of primers and probes. HBsAg secretion was measured using colorimetric-based ELISA DS-IFAHBsAg-0.01 kit. Target gene activation levels and antiviral activity of dSaCas9-p300 RNPs were compared with transient transfection and lentiviral transduction.

**Results: **dSaCas9-p300 RNPs resulted in transient (< 24 h) and robust (> tenfold) activation of A3A and A3B genes. Compared to transient transfection and lentiviral transduction, intracellular delivery of dSaCas9-p300 RNPs more effectively suppressed HBV RNA and DNA levels, and resulted in substantial decline in HBsAg levels.

**Conclusions: **In this work, for the first time we demonstrate the ability of dCas9 RNPs to potently activate target gene expression, resulting in significant decline in HBV replication intermediate levels. In fact, this is the first indication that dCas9 RNPs can effectively function in a transient, single delivery mode. We also compare dCas9 RNPs with transient transfection and lentiviral transduction methods, concluding that transient and robust functioning of RNPs is more effective than transient transfection and is on par with long-term lentiviral transduction.

**Acknowledgements: **This work was funded by RFBR-DFG grant 20-515-12010 and GL 595/9-1; and RFBR grant 20-015-00442.

**References**

1. Lucifora J, Xia Y, Reisinger F, Zhang K, Stadler D, Cheng X, Sprinzl MF, Koppensteiner H, Makowska Z, Volz T, Remouchamps C, Chou W-MW-M, Thasler WE, Huser N, Durantel D, Liang TJ, Munk C, Heim MH, Browning JL, Dejardin E, Dandri M, Schindler M, Heikenwalder M, Protzer U (2014) Specific and nonhepatotoxic degradation of nuclear hepatitis B virus cccDNA. Science (80-) 343:1221–1228. https://doi.org/10.1126/science.1243462

2. Brezgin S, Kostyusheva A, Bayurova E, Volchkova E, Gegechkori V, Gordeychuk I, Glebe D, Kostyushev D, Chulanov V (2021) Immunity and Viral Infections: Modulating Antiviral Response via CRISPR–Cas Systems. Viruses 13:1373.

3. Brezgin S, Kostyusheva A, Kostyushev D, Chulanov V (2019) Dead Cas Systems: Types, Principles, and Applications. Int J Mol Sci 20:6041

### O22. Update on hepatocellular carcinoma breakthroughs: do hepatocellular carcinomas have an achilles’ heel?

#### Laetitia Gerossier, Janet Hall, Alexia Paturel, Isabelle Chemin*

##### CRCL, INSERM U1052, CNRS 5286, Université de Lyon, 151 cours A Thomas, 69424, Lyon, France

###### Presenting author: Isabelle Chemin (isabelle.chemin@inserm.fr)

*Infectious Agents and Cancer* 2022, **17(Suppl 1)**: O22

**Background: **Hepatocellular carcinoma (HCC) is the most common primary liver cancer and the fourth-leading cause of cancer-related mortality worldwide. HCC has been increasing in incidence since the 1980s,^3^ and is now the fastest-rising cause of cancer-related death, with an estimated 1,284,252 deaths predicted between 2018 and 2040 worldwide. The majority of HCC occurs in the setting of chronic liver disease, with the most common risk factors being chronic hepatitis B virus (HBV) worldwide and hepatitis C virus-related cirrhosis and nonalcoholic steatohepatitis in the Western world. The estimated 5-year survival rate for HCC is only 18%, which is driven by a large proportion of patients being diagnosed at advanced stages when curative options are not feasible, as well as underuse of curative therapies among patients detected at early stages. Poly(ADP-ribose) polymerase inhibitors (PARPi) combined with radiotherapy is a promising cancer treatment. To address this combination’s applicability to hepatocellular carcinoma (HCC) the sensitivity of liver cell models to Veliparib and Talazoparib ± radiation exposure was determined. These lines expressed or not the hepatitis B virus X protein (HBx) known to deregulate cellular DNA damage repair via SMC5/6 degradation. Additionally, PARP expression profiles and DNA damage levels using the surrogate marker gammaH2AX were assessed in HCC tissues.

**Methods: **Cell cytotoxicity was measured by clonogenic survival or cell growth and the DNA damage response using immunological techniques in Hep3B, PLC/PRF/5, HepG2- and HepaRG-derived models. Transcriptome changes due to HBx expression vs SMC6 loss were assessed by RNA sequencing in HepaRG-derived models. PARP transcripts (qPCR) and PARP1, H2AX and gammaH2AX protein (RPPA) levels were compared in control liver vs HBV-, HCV-, alcohol- and NASH-associated HCC (Tumor/Peri-Tumor) tissues.

**Results: **PARPi cytotoxicity was significantly enhanced when combined with X-rays (2 Gy) with the greater impact seen with Talazoparib. HBx expression significantly lowered survival, probably driven by SMC5/6 loss based on the transcriptome analysis and higher DNA damage levels. PARP1 and PARP2 transcript levels were significantly higher in Tumor than Peri-Tumor and control tissues with PARG transcripts higher in all except NASH-associated Tumor tissues. The HBV/HCV/alcohol-associated Tumor tissues studied had reduced H2AX but higher gammaH2AX protein levels providing evidence of increased DNA damage during liver disease progression.

**Conclusions: **These proof-of-concept experiments support PARPi alone or combined with radiotherapy, particularly for HBV-associated HCC, that warrant further investigation.

### O23. Evaluation of metabolic inhibitors as complements to virotherapy of solid tumors

#### Sofia Nefedorova*, Anastasia Lipatova, Alexander Ivanov

##### Engelhardt Institute of Molecular Biology, Russian academy of sciences, Moscow, Russia

###### Presenting author: Sofia Nefedorova (sofianefedorova@yandex.ru)

*Infectious Agents and Cancer* 2022, **17(Suppl 1)**: O23

**Background: **Increased aerobic glycolysis in cancer cells, known as the Warburg effect, is observed in a variety of malignant tumors. This metabolic alteration is considered as a characteristic biochemical symptom of cancer cells and seems to be associated with mitochondrial dysfunction or respiration injury. Moreover, such adaptations are associated with certain tumor-associated mutations (like in K-Ras gene) which gives an opportunity to use metabolic inhibitors to augment action of oncolytic viruses. And combining metabolic inhibitor with viral anti-cancer therapy is a potential approach to treating cancer. Therefore, we attempted to find out an optimal combination of the metabolic inhibitor and the oncolytic enterovirus.

**Materials and methods: **A panel of model and primary tumor cell lines (A172 ATCC number CRL-16205522, 4434, u251MG) were treated with 2-deoxy-D-glucose, an analogue of pharmacologic drug fluoroglucose, at different concentrations (1–20 mM) and infected with Coxsackievirus B5, in wide range of MOI (from 0.0001 to 100) for 2–24 h. Cell viability was assessed by Celltracker dye (Thermo Fisher Scientific).

**Results: **We have shown synergetic cytopathic effect of 2-deoxy-D-glucose pretreatment and viral infection on primary glioblastoma 5522 and 4434, model glioblastoma U251 and A172, as well as Hela cells: the effect of virotherapy was more pronounced after 2-deoxy-D-glucose pretreatment. The highest effect was achieved in treatment of the primary glioblastoma cells with 2 mM 2-deoxyglucose simultaneously with virus infection. In this case, the drug increased oncolytic activity of the virus by tenfold. Interestingly, the effect was cell line-specific: the drug did not enhance cell death in three other tested cell lines. Other metabolic inhibitors are also under evaluation.

**Conclusions: **To verify this effect, in vivo models should be used (immunodeficient mice with primary glioma xenografts). In addition, it’s necessary to evaluate metabolic changes in cells during viral infection using Extracellular flux analyzer Seahorse (Agilent) to show changes in glycolysis rate and understand precisely which other metabolic inhibitors could be used for enhancement of viral oncolysis effectiveness.

**Acknowledgements: **The study was supported by the Russian Science Foundation grant No 19-14-00197.

### O24. Immunogenicity of combined DNA/protein anti-SARS-COV-2 vaccine

#### Maria B. Borgoyakova*, Yulia A. Merkuleva, Larisa I. Karpenko, Dmitry N. Shcherbakov, Andrey P. Rudometov, Ekaterina V. Starostina, Daniil V. Shanshin, Natalya V. Volkova, Anastasiya A. Isaeva, Valentina S. Nesmeyanova, Ekaterina A. Volosnikova, Alexey M. Zadorozhny, Lyubov A. Orlova, Anna V. Zaikovskaya, Oleg V. Pyankov, Alexander A. Ilyichev.

##### State Research Center of Virology and Biotechnology "Vector" of Rospotrebnadzor

###### Presenting author: Maria B. Borgoyakova (borgoyakova_mb@vector.nsc.ru)

*Infectious Agents and Cancer* 2022, **17(Suppl 1)**: O24

**Background: **The continuing COVID-19 pandemic requires development of the new safe, effective and affordable anti-coronavirus vaccines. We have designed the original candidate vaccine that combines recombinant protein and DNA vaccine in a self-assembled virus-like particles. These VLPs contain DNA-vaccine pVAX-S, encoding full-size S protein of SARS-CoV-2 as a core covered with polyglucin:spermidine with the recombinant RBD protein on its surface.

**Material and methods: **Following in vitro confirmation and characterization of DNA-vaccine candidate, pVAX-S has been enveloped into a conjugate of the RBD protein, polyglucin and spemidine or into a polyglucin:spermidine conjugate for one of the control group. Verification of the complexation has been carried out before each immunization. The samples have been examined using electron microscopy; the mobility of the complexes has been analyzed using a 1% agarose gel. The humoral and cellular immune responses of the combined DNA/protein vaccine have been evaluated in BALB/c mice. Mice have been immunized twice with three weeks’ interval with intramuscular injection of the 100 µg DNA and 100 µg protein per dose. The equivalent doses of the DNA pVAX-S or RBD protein have been used for immunization of the control mice groups. Blood and splenocytes have been harvested ten days after the second immunization for ELISA, virus neutralization, ELISpot and ICS analysis.

**Results: **Immunization with combined DNA/protein vaccine resulted in significant stimulation of the S- and RBD-specific antibodies which had high live virus neutralizing activity versus immunization with DNA or protein alone (at least tenfold as high as in the control groups). As for T-cell response, the highest levels of IFN-γ producing cells including CD4 + and CD8 + T-lymphocytes were shown in the group immunized with plasmid pVAX-S alone. The levels of IFN-γ producing T-cells in the group received the combined vaccine were two times less than in the DNA group while the group immunized with protein showed no stimulation of cellular immunity at all. Significance between groups was calculated using nonparametric Mann–Whitney method by GraphpadPrism 6.0 software.

**Conclusion: **This study demonstrates that combining different types of immunogens, DNA and protein, in one construction leads to synergistic enhancement of the humoral immune response including virus-neutralizing activity. Combined DNA/protein vaccine induces a significant response of virus-specific CD8 + and CD4 + T-lymphocytes producing IFNγ in BALB/c mice.

The results suggest that the pVAX-S enveloped into the polyglucin-spermidine-RBD conjugate can be considered as a promising platform for developing anti-SARS-CoV-2 vaccine.

**Acknowledgements: **The study was conducted under the state assignment of FBRI SRC VB “Vector”, Rospotrebnadzor.

### O25. Recombinant viral vectors as adjuvants and modulators of immune response for cancer treatment

#### Anna Zajakina

##### Latvian Biomedical Research and Study Centre, Riga, Latvia

###### Presenting author: Anna Zajakina (anna.zajakina@gmail.com)

*Infectious Agents and Cancer* 2022, **17(Suppl 1)**: O25

Viruses are natural mobile genetic elements that have effective properties of transferring genetic information in living organisms. Due to their ability to induce potent multilateral immune responses, viral vectors have recently received renewed attention as a platform of choice to develop next generation vaccines and immunotherapy adjuvants. In the field of cancer immunology, the discovery of tumor-specific neoantigens to which there is no self-tolerance and the approval of effective immune checkpoint inhibitors has led to the clinical development of novel vector-based cancer therapies. The use of cytokines as vaccine adjuvants has a unique value in obtaining the appropriate immune response and in ensuring a protective outcome, especially in low/non-responding and immunosuppressed individuals. Previous studies indicate that cytokines can influence the generation of a particular antibody isotype and may represent a tool to enhance systemic T-cell immune response, as well as to induce stronger immunological memory. The vector-based delivery of immune-modulating cytokines potentially can help to overcome the limitations of cytokine direct administration, such are the toxicity and the short half-life of cytokines in vivo.

Among different vectors, the recombinant vaccines based on alphaviruses have demonstrated both prophylactic and therapeutic efficacy in pre-clinical studies. Additionally, alphaviral vectors have proven their safety in Phase I clinical trials in melanoma and kidney carcinoma patients. Recently, we have designed a set of Semliki Forest virus (SFV) and Sindbis virus (SIN) vectors producing proinflammatory cytokines and peptides (IL12, IFNγ, TNFα, anti-TGF-beta peptides), and confirmed their antitumour potential in mouse breast cancer model. The main focus of our study is the remodelling of immunosuppressive tumour environment to avoid chemoresistance and immunotherapy escape scenario.

We showed that virus-derived IFNγ synergised with a TLR2/1 agonist to induce nitric oxide production in macrophages as a strong proinflammatory signal of anti-tumour macrophage programming leading to tumour inhibition. Importantly, that the SFV vector itself inhibited tumour growth and led to intratumoral increase of CD8 T cells and a decrease of myeloid cell populations. Furthermore, we showed polarization of macrophages to M1 phenotype, which increases the potency of vaccines through maximizing the antigen presentation by MHCII upregulation in M1 macrophages.

The biological features of alphaviruses have made them ideal tool for vaccine adjuvant development. A broad host range, direct targeting of dendritic cells, lack of genome integration, cytoplasmic RNA amplification and high-level antigen expression are important advantages for the application of alphaviruses as genetic complementary vaccines.

### O26. Macrophage 3D cultivation as a promising model to study the induction of innate immune response against viral infections and cancer

#### Ksenija Korotkaja*, Juris Jansons, Anna Zajakina

##### Latvian Biomedical Research and Study Centre, Riga, Latvia

###### Presenting author: Ksenija Korotkaja (ksenija.korotkaja@biomed.lu.lv)

*Infectious Agents and Cancer* 2022, **17(Suppl 1)**: O26

**Background: **Macrophages are involved in non-specific immune response and, as a result, in progression of viral infections and cancer. Traditionally macrophages are divided in two polarization states: pro-inflammatory M1 and anti-inflammatory M2. However, macrophages possess high plasticity and have been shown to express more polarization states depending on activation stimuli [1]. Conventional cell cultivation in monolayer does not represent organism architecture and cell–cell interactions. 3D systems for cell cultivation can improve predictability and accuracy of in vitro studies [2]. In previous studies the 3D model for co-cultivation of macrophages and cancer cells was presented [3]. The aim of this study is to comprehensively characterize the macrophage molecular profile in 3D cultivation model.

**Materials and methods: **In this study we have used ultra-low attachment 96-well plate to achieve 3D conditions. Murine Bone Marrow-Derived Macrophages (BMDMs) were obtained from BALB/c mice. Macrophages were polarized in vitro by adding 50 ng/mL IFNγ and 100 ng/mL Pam3SCK4 to induce M1 phenotype and 20 ng/mL IL-4 to induce M2. Flow cytometry was performed to determine changes in intracellular and extracellular marker expression, ELISA was performed to investigate secreted cytokine profile. Chemokine profile was determined using Luminex assay.

**Results: **In this study we have developed and characterized a 3D in vitro model for studying different macrophage phenotypes. We have determined the secretome, surface marker profile and secreted chemokine profile of 3D cultivated M0, M1 and M2 macrophages. The results show that macrophages can be successfully cultivated and polarized in the 3D model. Furthermore, we have investigated the plasticity of macrophages by establishing a re-polarization model. We revealed a rapid alteration of the macrophage polarization state after the change of added polarization stimuli. However, re-polarized macrophages start to express markers of new phenotype while continuing to exhibit the features of previous phenotype.

**Conclusions: **In summary, the characterized 3D in vitro model is a promising method for investigation of immune cell response to different stimuli. Furthermore, our findings suggest that re-polarized macrophages obtain mixed phenotype. The developed 3D model can be applied for programming of macrophage response as an immunotherapy approach in treatment of infectious diseases and cancer.

**Acknowledgements: **This study was supported by Project No: lzp-2020/2-0376 “Pathogenicity of human Hepatitis C virus relying on enzymatic properties of viral RNA-dependent RNA-polymerase” and Project No: lzp-2018/1-0208 “Functional programming of tumor-associated macrophages with viral immunotherapy vectors in breast cancer model”.

**References**

1. Mills, C. D. M1 and M2 Macrophages: Oracles of Health and Disease. Crit Rev Immunol 2012, 32 (6), 463–488. https://doi.org/10.1615/critrevimmunol.v32.i6.10

2. Boutin, M. E.; Voss, T. C.; Titus, S. A.; Cruz-Gutierrez, K.; Michael, S. Ferrer M. A high-throughput imaging and nuclear segmentation analysis protocol for cleared 3D culture models. Sci Rep 2018, 8. https://doi.org/10.1038/s41598-018-29169-0

3. Trofimova, O.; Korotkaja, K.; Skrastina, D.; Jansons, J.; Spunde, K.; Isaguliants, M.; Zajakina, A. Alphavirus-Driven Interferon Gamma (IFNg) Expression Inhibits Tumor Growth in Orthotopic 4T1 Breast Cancer Model. Vaccines 2021, 9, 1247. https://doi.org/10.3390/vaccines9111247

### O27. SARS-COV-2 RBD conjugated with polyglucinum-spermidine and dsRNA elicits strong immune response in mice

#### Yulia A. Merkuleva*, Dmitry N. Shcherbakov, Ekaterina A. Volosnikova, Tatiana I. Esina, Maria B. Borgoyakova, Anastasiya A. Isaeva, Valentina S. Nesmeyanova, Natalya V. Volkova, Daniil V. Shanshin, Svetlana V. Belenkaya, Andrey P. Rudometov, Larisa I. Karpenko, Anna V. Zaykovskaya, Oleg V. Pyankov, Alexander A. Ilyichev

##### State Research Center of Virology and Biotechnology “Vector”, Rospotrebnadzor, World-Class Genomic Research Center for Biological Safety and Technological Independence, Federal Scientific and Technical Program on the Development of Genetic Technologies, Koltsovo, Novosibirsk region, Russia

###### Presenting author: Yulia A. Merkuleva (j.a.merkulyeva@gmail.com)

*Infectious Agents and Cancer* 2022, **17(Suppl 1)**: O27

**Background: **Despite the rapid development and approval of several COVID-19 full-length Spike (S) protein vaccines, there is a need for safe, potent, and high-volume vaccines. Given the predominance of the production of neutralizing antibodies that target RBD after natural infection or vaccination, it makes sense to choose RBD as the only immunogen in the vaccine [1]. However, due to its small size, RBD is relatively poorly immunogenic. The search for new adjuvants for RBD-based vaccine formulations is considered a good strategy for enhancing its immunogenicity. The aim of this study was to assess the immunogenicity of SARS-COV-2 RBD conjugated with polyglucinum-spermidine (PGS) in mice model.

**Materials and methods: **A DNA fragment encoding RBD (a.a. 320 V–542 N) was codon-optimized and synthesized. Spike signal sequence was added upstream of the N-terminus, and 10His-tag sequence downstream C-terminus of RBD, and cloned into pVEAL2 integrative plasmid vector. CHO-K1 cell line was used to stable RBD production. Recombinant proteins were purified by affinity and ion exchange chromatography. Dextran (MW 40,000) was activated with sodium periodate, purified by gel filtration and, mixed with RBD protein solution. Thereafter, the spermidine solution and sodium borohydride were added, and after incubation, the resulting RBD-PGS conjugate was purified and mixed with dsRNA. The efficiency of the RBD-PGS + dsRNA complexes formation was assessed by the change in the electrophoretic mobility of DNA in 1% agarose gel. Female BALB/c mice were randomly divided into 5 groups (n = 10) and immunized intramuscularly twice at a 2-week interval with 50 µg of RBD, RBD conjugated with PGS or PGS + dsRNA, RBD with Al(OH)3. The control group received PBS. Two-week after booster injection mice sera were accessed in ELISA and virus neutralization test using SARS-CoV-2 coronavirus strain nCoV/Victoria/1/2020. Mice splenocytes were analyzed using Intracellular cytokine staining (ICS) assay to assess T-cell immune response.

**Fig. 1 (Abstract O27):**
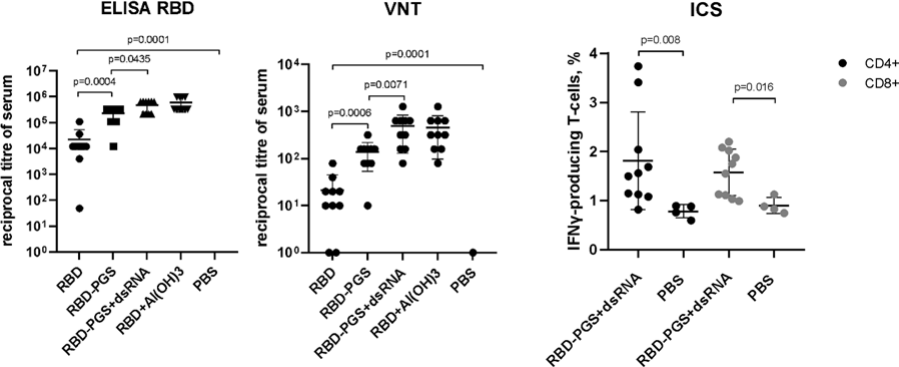
Immune response to conjugated RBD in mice: **a** Sera IgG specificity to recombinant RBD in ELISA; **b** Sera neutralization titers against SARS-CoV-2/Victoria/1/2020; **c** ICS assay for antigen-specific CD4 + and CD8 + T-cell secretion of IFN-γ in mice splenocytes. The data were plotted as the mean ± SD. Two-tailed Mann–Whitney U test P-values indicate statistical significance

**Results: **The comparative analysis of mice sera RBD-specific IgG and neutralizing antibody titers showed that PGS, PGS + dsRNA, and Al(OH)3 enhance specific humoral response in animals. There was no significant difference between the groups immunized with RBD-PGS + dsRNA and RBD with Al(OH)3. However, RBD-specific IgG titers were 27-fold, and neutralizing antibody titers were threefold higher in the group immunized with RBD-PGS + dsRNA than the group immunized with RBD + PGS (Fig. 1a, b).

Next, ICS assay for antigen-specific CD4 + and CD8 + T-cell secretion of IFN-γ in mice splenocytes showed that RBD-PGS + dsRNA provides a specific cellular immune response in animals (Fig. 1c).

**Conclusions: **A comparative immunogenicity assessment of RBD conjugated with PGS or PGS + dsRNA, and RBD in combination with Al(OH)3 revealed that PGS + dsRNA significantly enhances the specific humoral and cellular immune response to RBD protein in mice. Therefore, we suggest that the conjugation of immunogen with PGS + dsRNA can be an alternative to adjuvants for RBD-based subunit vaccine formulation.

**Acknowledgments: **This study was supported by the Ministry of Science and Higher Education of the Russian Federation (agreement № 075-15-2019-1665).

**References**

1. Kleanthous, H., Silverman, J.M., Makar, K.W. et al. Scientific rationale for developing potent RBD-based vaccines targeting COVID-19 // npj Vaccines. 2021. V. 6(1). P. 128.

## Poster session

### P1. Concordance of the Xpert hepatitis B viral load test and conventional quantitative PCR in detecting and quantifying viremia using stored plasma and dried blood spot samples in West Africa

#### Amie Ceesay^1,3*^, Gibril Ndow^1,2^, Yusuke Shimakawa^4^, Alpha Omar Jallow^1^, Hady Nyang^1^, Baboucarr Bittaye^1^, Francis Mendy^1^, Ousman Secka^1^, Umberto D’Alessandro^1^, Mark Thursz^2^, Isabelle Chemin^3^, Maud Lemoine^2^.

##### ^1^Medical Research Council (MRC) Unit The Gambia at the London School of Hygiene & Tropical Medicine, Fajara, The Gambia; ^2^Department of Metabolism, Digestion & Reproduction, Imperial College London, UK; ^3^INSERM U1052, CNRS UMR5286, Centre de Recherche en Cancérologie, Université Claude Bernard, Lyon, France; ^4^Unité d'Épidémiologie des Maladies Émergentes, Institut Pasteur, Paris, France

###### Presenting author: Gibril Ndow (amceesay10@gmail.com)

*Infectious Agents and Cancer* 2022, **17(Suppl 1)**: P1

**Background: **The new Xpert hepatitis B virus (HBV) viral load test (Cepheid, USA) provides an opportunity to scale up and decentralise hepatitis B diagnosis, treatment and monitoring in resource-limited settings where HBV is endemic and molecular diagnostics to quantify HBV DNA are scarce. The aim of this study was to assess the concordance between the new Xpert HBV viral load test and the conventional quantitative PCR (Abbott, USA) in quantifying HBV viremia using stored plasma and dried blood spot (DBS) samples.

**Materials and methods: **Paired plasma and DBS (Whatman 903 Protein Saver cards) samples were collected from adults with chronic HBV infection followed up in the PROLIFICA (Prevention of Liver Fibrosis and Cancer in Africa) programme in The Gambia. Samples were stored in -80C for at least 12 months before analysis.

For each plasma sample, HBV DNA was quantified using both realtime qPCR (Abbott, limit of detection 26 IU/mL) and Xpert (limit of detection 10 IU/mL). For DBS, HBV DNA was quantified using Xpert.

Concordance of the Xpert HBV test and the conventional qPCR was assessed by kappa statistics. The agreement between these two assays was assessed using the Bland–Altman plot. The concordance of HBV viremia between paired plasma and DBS samples was also assessed by kappa statistics.

**Results: **We analysed 266 stored plasma samples from patients at various hepatitis B disease stages. Of these, HBV viral load results were concordant between the Xpert and the conventional qPCR: 130 (48.9%) and 63 (23.7%) were detectable and undetectable by both assays, respectively. The results were discordant in 71 (26.7%) samples that were detectable by Xpert but not qPCR, and 2 (0.8%) samples that were detectable by qPCR but not Xpert. In 110 samples with quantified viral load results from both assays, the mean difference between the two assays was 0.316 log IU/mL (95% limits of agreement − 1.189, 1.820) with only 6/110 results (5.45%) being outside the limits of agreement (Fig. 1), indicating good agreement.

**Fig. 1 (Abstract P1):**
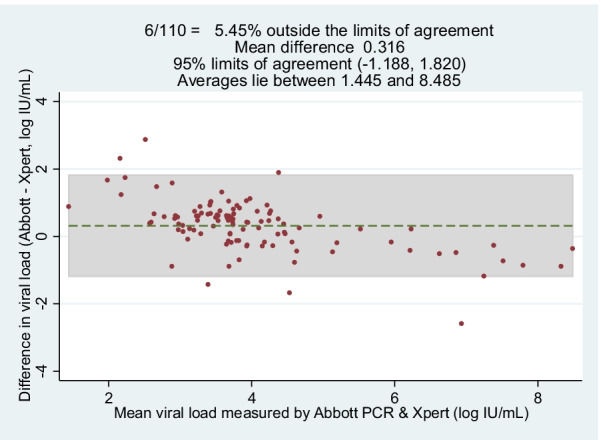
Bland-Altmann plot of Xpert HBV minus Abbott qPCR (vertical axis) against mean of Xpert HBV and Abbott qPCR (horizontal axis) for samples with detectable viral load (n = 110)

From 91 paired plasma and DBS samples analysed by Xpert assay, the mean bias between plasma and DBS was statistically significant at + 1.831 log IU/mL (95% limits of agreement: 0.660–3.001). Bias was smaller at lower viral loads. The kappa statistic was 0.10 indicating very poor agreement between results. After excluding samples with error or invalid result by either sample type (n = 40), kappa statistic was 0.34 indicating a poor agreement.

**Conclusion: **The Xpert HBV DNA test provides a reliable alternative for detecting and quantifying HBV DNA viral load using stored plasma samples. However, the assay is not reliable for detecting viremia from DBS samples.

### P2. Early hypocoagulation predicts severe acute respiratory distress syndrome in unvaccinated patients with COVID-19 related pneumonia

#### Denis S. Baranovskii*^1,2^, Ilya D. Klabukov^2^, Olga A. Krasilnikova^2^, Alexey V. Lyundup^1^, Mikhail E. Krasheninnikov^1^, Maxim V. Balyasin^1^, Michael I. Danilevskiy^1^

##### ^1^Research and Educational Resource Center for Cellular Technologies, Peoples’ Friendship University of Russia, Moscow, Russia; ^2^Department of Regenerative technologies and Biofabrication, National Medical Research Radiological Center, Obninsk, Russia

###### Presenting author: Denis S. Baranovskii (doc.baranovskii@unibas.ch)

*Infectious Agents and Cancer* 2022, **17(Suppl 1)**: P2

**Background: **Microcirculation abnormalities associated with coagulopathy may lead to acute respiratory distress syndrome (ARDS) in COVID-19 patients. In the present study, we aimed to identify on-admission coagulation parameters that may early indicate further respiratory failure or severe acute respiratory distress syndrome requiring treatment in an intensive care unit.

**Materials and methods: **We investigated differences in prothrombin time (PT) and international normalized ratio (INR) as well as in Fibrinogen and D-dimer levels between unvaccinated COVID-19 patients who had been transferred to an ICU within two weeks after admission (n = 82) and COVID-19 patients with stable course of the disease (n = 74).

**Results and discussion: **Multiple comparisons showed statistically significantly prolonged PT on admission in ICU-transferred COVID-19 patients (14.15 s, median, CI 95% 13.4 ÷ 14.9) compared to the stable COVID-19 patients (13.25 s, median, CI 95% 12.9 ÷ 13.6) (p-value = 0.0005). On-admission D-dimer, fibrinogen and INR levels did not statistically significantly differ between ICU-transferred COVID-19 patients and stable COVID-19 patients. We suggest that appearance of an incipient hypocoagulation could early indicate severe complications of COVID-19 requiring transfer to ICU.

### P3. Enhanced motility of adenocarcinoma cells expressing HIV-1 protease

#### Ekaterina Bayurova^1,2*^, Oleg E. Latyshev^1*^, Athina Kilpeläinen^3^, Stefan Petkov^3^, Anastasiya Latanova^1,4^, Olesia Eliseeva^1^, Olga A. Smirnova^4^, Dmitry Kostyushev^5,6^, Anastasiya Kostyusheva^5^, Tatiana Gorodnicheva^7^, Ilze Fridrihsone^8^, Svetlana Gebrila^8,9^, Juris Jansons^8,10^, Elizaveta Starodubova^3,4^, and Maria G. Isaguliants^1,2,3,8^

##### ^1^N.F. Gamaleya Research Center for Epidemiology and Microbiology, 123098 Moscow, Russia; ^2^Chumakov Federal Scientific Center for Research and Development of Immune-and-Biological Products of Russian Academy of Sciences, 108819 Moscow, Russia; ^3^Department of Microbiology, Tumor and Cell Biology, Karolinska Institutet, 17177 Stockholm, Sweden; ^4^ Engelhardt Institute of Molecular Biology, Academy of Sciences of the Russian Federation, Moscow, Russia; ^5^ National Medical Research Center of Tuberculosis and Infectious Diseases, Ministry of Health, 127994, Moscow, Russia; ^6^ Scientific Center for Genetics and Life Sciences, Division of Biotechnology, Sirius University of Science and Technology, 354340 Sochi, Russia; ^7^Evrogen, Moscow, Russia; ^8^ Riga Stradins University, Riga, Latvia; ^9^ Paul Stradins University Hospital, Riga, Latvia; ^10^ Biomedical Research and Study Center, Riga, Latvia

###### Presenting author: Ekaterina Bayurova (bayurova_eo@chumakovs.su)

*Infectious Agents and Cancer* 2022, **17(Suppl 1)**: P3

**Background: **HIV-1 infection leads to increased morbidity and mortality from different forms of cancer, despite the background of successful antiretroviral treatment (ART). In part, this is due to viral integration and DNA damage, and in part, to direct carcinogenic effects of HIV-1 proteins [1]. Several proteins, as gp120, Tat, Rev, Nef, p17, induce oxidative stress, and have direct oncogenic effects on the expressing as well as exposed uninfected cells due to their release into extracellular space. Lately, we identified another HIV protein that induces ROS, is found in extracellular space and increases tumorigenic and metastatic potential of tumor cells—HIV-1 reverse transcriptase [1]. Here, we inquired if there exist additional unrelated mechanisms of HIV-1 induced oncogenesis which involve other HIV-1 proteins, and focused on an enzyme vital for virus infectivity, HIV-1 protease. The aim of this study was to evaluate the effects of HIV-1 protease expression on epithelial cells in vitro and in vivo.

**Materials and methods: **Consensuses for PR of HIV clade A FSU_A strain PR_A (PR_A) was designed, synthesized and cloned into pET15b for prokaryotic and pVax1 for eukaryotic expression. PR_A was expressed in E coli, and used to immunize rabbits following standard protocol with permission of the Ethical Committee of Gamalyea Research Center. Inactivation mutation (D25N) allowing detectable eukarytic expression (PR_Ai) and primary drug resistance mutations frequent in the FSU-A strain (M46I, I54V, V82A; PR_A3) were introduced by site-directed mutagenesis (Evrogen) resulting in plasmids encoding PR_A, PR_Ai, PR_A3 and PR_A3i, respectively. Expression of PR variants in HEK293 cells was confirmed by Western blotting with rabbit anti-PR_A antibodies. Partial inactivation of PR_A in lysates of cells transfected with D25N compared to non-mutated PR was confirmed by FRET assay (SensoLyte 490, Anaspec). PR_A3i DNA was recloned into lentiviral vector pRRLSIN.PGK (Evrogen); lentiviral particles were generated and used to transduce 4T1luc2 cells (Calipers) to obtain 4T1luc2 subclones 4T1luc2_PR20.1 and 4T1luc2_PR20.2 encoding PR_A3i. Levels of reactive oxygen species (ROS) produced by 4T1luc2_PR20.1/PR20.2 were measured using 2’,7’-dichlorodihydrofluoresceine diacetate (DCFH2-DA). Lipid oxidation was evaluated by measuring the level of malondialdehyde (MDA) using HPLC with MS detection. Subclones were cultured with and without antioxidant N-acetyl cysteine (NAC; 5uM); expression of *E-cadherin*, *N-cadherin*, *Vimentin*, *Snail* and *Twist* were analyzed by RT_PCR. Wound healing assay (WHA) was performed with or without NAC, and assessed using light microscopy (Leica, Wetzlar, Germany). BALB/C mice were subcutaneously implanted with 10(4) 4T1luc2_PR20.1 or 4T1luc2_PR20.2 cells (n = 6 each). Tumor growth was monitored morphometrically and by bioluminescence imaging (BLI; Spectrum CT with Living Image 4.4 software). At experimental end-point by day 20, mice were sacrificed; tumors and internal organs were excised, and monitored by ex vivo BLI. Half of the tumors were homogenized, RNA was extracted and subjected to RT-PCR to confirm PR mRNA expression. The other tumor half and murine organs were dehydrated, paraformaldehyde fixed and paraffin-embedded, FFPE blocks were sliced, H&E stained and subjected to histological analysis. Results were analyzed by Kruskal–Wallis and two-way ANOVA tests, p < 0.05 was considered significant.

**Results: **PR variants expressed from the synthetic genes were specifically stained with rabbit anti-PR antibodies. D25N mutation caused 60–70% decrease of PR activity. Introduction of DR and inactivation mutations increased the level of PR expression 3-5 fold. PR_Ai3 providing the highest level of expression was recloned into lentiviral vector used to transduce murine adenocarcinoma 4T1luc2 cells. Subclones 4T1luc2_PR20.1 and PR20.2 were obtained expressing PR_Ai3 mRNA. Levels of ROS in 4T1luc2_PR20.2 and parental cells were comparable, whereas 4T1luc2_PR20.1 produced elevated levels of ROS, resulting in increased lipid peroxidation. In WHA, PR-expressing subclones migrated faster than the parental cells, specifically at the stage of wound closure. Migration rate of PR-expressing 4T1luc2 cells was further increased by treatment with antioxidant N-acetyl-cysteine (NAC). NAC treatment of PR_A-expressing subclones decreased expression of EMT factors below the levels observed for parental cells. In PR20.2 subclone it also led to five-fold increase in expression of *N-cadherin*. In BALB/c mice, both subclones formed larger tumors than parental cells, from day 12 4T1luc2_PR20.2, inducing lower ROS, grew more aggressively generating higher BLI signals and tumors of larger size (p < 0.05). PR20.2-tumors expressed PR mRNA at 4-times higher level than tumors formed by PR20.1. PR20.2-implanted mice had higher number of metastases, observed mainly in lungs. Histological evaluation revealed high grade tumors with low differentiation for both subclones.

**Discussion: **High motility is an important factor determining metastatic activity of cancer cells. In HIV-1 infection, high capacity for migration and trafficking contributes to efficient spread of infected cells [2]. Here, we observed that expression of HIV-1 PR caused enhanced motility of expressing cells in vitro and increased metastatic activity in vivo, specifically for a subclone PR20.2 with higher level of PR expression. Inactivated by D25N mutation to increase expression PR_Ai retained residual protease activity (30%). In adenocarcinoma cells, treatment with proteases is known to promote cell migration unrelated to expression of EMT markers [3]. With this, increased motility of PR20.2 cells in vitro and enhanced tumor growth and increased metastatic activity of PR20.2 in vivo, is not due to oxidative stress and induction of EMT, since PR20.2 cells produced lower levels of ROS than PR20.1 cells and exhibited same levels of expression of EMT factors as the parental cells. Instead, we hypothesize that it is related to protease activity that may increase cell motility similar to the effect of MMP-2/gelatinase. In lines with this, highly expressing PR20.2 cells had higher metastatic activity in vivo than PR20.1 subclone with a lower level of PR expression. Proteases could be completely inactivated by oxidation of Cys leading to formation of disulfide bonds [4]. Antioxidant/NAC treatment reduces disulfide bonds and restores PR activity [5]. Hence, NAC treatment of PR-expressing clones could be expected to increase PR activity and cell motility, as was observed for highly expressing PR20.2 cells.

**Conclusions: **HIV-1 protease induces an increased cell migration (motility) in vitro and in vivo not associated with the induction of oxidative stress and not consistently associated with the expression of the EMT proteins, i.e. independently of EMT, but due to intrinsic HIV-1 PR activity. This phenomenon may stand behind high motility of HIV-1 infected cells that contributes to efficient viral spread in the infected subjects.

**Acknowledgements: **RFBR 19_04_01034; 17_04_0583; RFBR grant 20-015-00442 to D.K., A.K., VACTRAIN #692293 and NAWA to RSU.

**References**

1. Isaguliants M et al. Cancers (Basel). 2021 Jan 15;13(2):305. https://doi.org/10.3390/cancers13020305;

2. Murooka T et al. Nature. 2012 Oct 11;490(7419):283–7. https://doi.org/10.1038/nature11398. Epub 2012 Aug 1.

3. McNair K et al. Cancer Biol Ther. 2019;20(3):349–367. https://doi.org/10.1080/15384047.2018.1529109.

4. Davis DA et al. J Virol. 1999 Feb;73(2):1156–64. https://doi.org/10.1128/JVI.73.2.1156-1164.1999.

5. Aldini G et al. Free Radic Res. 2018 Jul;52(7):751–762. https://doi.org/10.1080/10715762.2018.1468564.

### P4. Primary hepatocytes of common marmosets *Callithrix jacchus* as a platform to create cell lines expressing viral proteins and human tumor-associated antigens

#### Ekaterina Bayurova^1 2^, Dmitry Kostyushev^3,4^, Anastasiya Kostyusheva^3^, Sergey Brezgin^3,4^, Sergei I. Belikov^1,5^, Darya Avdoshina^1^, Liliya P. Antonova^1^, Tatiana Gorodnicheva^6^, Maria G. Isaguliants^1,2,7,8^ and Ilya Gordeychuk^1,2*^

##### ^1^Chumakov Federal Scientific Center for Research and Development of Immune-and-Biological Products of Russian Academy of Sciences, 108819 Moscow, Russia; ^2^ National Research Center for Epidemiology and Microbiology named after NF Gamaleya, 123098 Moscow, Russia; ^3^ National Medical Research Center of Tuberculosis and Infectious Diseases, Ministry of Health, 127994, Moscow, Russia; ^4^ Scientific Center for Genetics and Life Sciences, Division of Biotechnology, Sirius University of Science and Technology, 354340 Sochi, Russia; ^5^ Department of Molecular Biosciences, The Wenner-Gren Institute, Stockholm University, S-106 91, Stockholm, Sweden; ^6^ Evrogen, Moscow, Russia; ^7^ Riga Stradins University, Riga, Latvia; ^8^ Department of Microbiology, Tumor and Cell Biology, Karolinska Institutet, 17177 Stockholm, Sweden

###### Presenting author: Ilya Gordeychuk (lab.gord@gmail.com)

*Infectious Agents and Cancer* 2022, **17(Suppl 1)**: P4

**Background: **Hepatotropic viruses such as HBV and HCV cause human infections with severe consequences for affected individuals and for health care system. There are only few cell culture models available for studying hepatotropic viruses. The majority of these models are of human origin. Lack of animal cell culture models leads to shortage of animal models for testing antiviral drugs and vaccines. Developing animal-based models of infection by hepatotropic viruses is urgently needed for studying basic viral biology, liver disease immunopathology and drug pre-clinical trials. Common marmoset (Callithrix jacchus, CM) is a well described and widely used animal model for testing antiviral strategies [1, 2] phylogenetically close to humans and with a unique immune system. CM-derived cell lines expressing viral proteins and tumor associated antigens (TAA) could serve as an attractive basis to design of CM-based models to study HBV and HCV infections and associated cancers. The aim of this study was to culture CM hepatocytes, characterize their properties and investigate possibility of their immortalization, in order to create CM-based cell lines expressing HBV, HCV proteins and TAA.

**Materials and methods: **Liver of CM fetus from a deceased animal was excised and placed into PBS supplemented with penicillin (100 U), streptomycin (100 mM) and amphotericin B (0.25 μg) (all Gibco). To obtain suspension of hepatocytes liver was perfused with solution I (2.5 mM EGTA; 1.25 mM N-Acetyl cysteine; 142 mM NaCl; 6.7 mM KCl; 10 mM HEPES-Na pH 7.55), digestion solution (29 mM NaCl; 2.9 mM KCl; 43.35 mM HEPES-Na pH 7.55; 0.21% Albumin; 0.32 mM CaCl2; 10% FBS; collagenase IV 1 mg/ml). Then, liver was homogenized in digestion solution using sterile syringe till full dissociation; digestion was terminated by stop solution (ice cold 20% FBS in PBS). Cell suspension was filtered through 70 μM cell strainers (Coring) and centrifuged 50 g 5 min 4 °C. The pellet was washed in PBS and resuspended in hepatocytes incubation medium containing DMEM 4.5 g/L glucose supplemented with Penicillin (100 U) and Streptomycin (100 mM); Hydrocortisone (0.1 μM); Sodium pyruvate (1 mM); L-Glutamine (146 mg); HEPES-Na pH 7.55 (15 mM) (all PanEco); FBS (10%, HyClone), and ITS + (Corning), and plated onto plates coated with rat Collagen I (Abcam). On late passages cells were cultured using DMEM/F12 culture medium (PanEco) with 100 U Penicillin, 100 mM Streptomycin and 10% FBS. Cell morphology was monitored using Axio Observer A1 microscope (Zeiss). Doubling time was calculated using formula recommended by ATCC [3]. For albumin detection cells were cultured in serum free media for 48 h and culture media was analyzed by SDS-PAAG electrophoresis and standard calibration curve. Semi-quantitative qRT-PCR was performed with specific primers and SYBR Green dye to analyze expression of albumin, transferrin, glucose-6-phosphate, keratin and tyrosine-aminotransferase genes. Lentiviral particles encoding rat telomerase reverse transcriptase (TERT) were obtained using pLV-PGK-TERT carrying synthetic TERT [4] and further used to transduce hepatocytes at multiplicity of infection 8. TERT genomic insertion was confirmed by PCR. TERT activity was measured using TRAP assay as described [5]. For clonogenic assay, 200 cells were seeded onto 60 mm collagen-coated culture dish and monitored for one month.

**Results: **Primary hepatocytes could be reproducibly obtained using the described protocol and cultured up to one month without detectable changes in either cell phenotype, or transcription of genes characteristic to hepatocytes. Albumin secretion level was 47 μg/mL. After one month, cells underwent spontaneous phenotype change and were cultured for the following 6 months without signs of cell crisis. Lentiviral transduction with TERT led to stabilization and shortage of doubling time but did not promote either colony formation or collagen-independent growth. TERT-transduced hepatocytes differed from parental cells in the TERT activity evaluated by TRAP assay.

**Conclusions: **We have generated and characterized primary hepatocytes of the common marmoset. CM hepatocytes could be stably cultured for at least one month, can be transduced with lentiviruses to express tumor associated antigens as TERT, and can serve as a platform to generate immortalized cell lines.

**Acknowledgements: **RFBR grant 2019-04-01034 to E.B., D.A., M.I. and I.G.; RFBR grant 20-015-00442 to D.K., A.K. and S. Brezgin; Latvian Science Fund LZP 2018/2-0308 to M.I on TERT-related part of the study; and Cooperation Project in the Baltic Sea Region Dnr 09344-2020 to S. Belikov.

**References**

1. Gordeychuk I et al. Vaccine. 2021 Nov 23:S0264-410X(21)01487-0. https://doi.org/10.1016/j.vaccine.2021.11.042

2. Mansfield K. Comp Med. 2003 Aug;53(4):383–92. PMID: 14524414.

3. ATCC Animal Cell Culture Guide Available at: https://www.atcc.org/resources/culture-guides/animal-cell-culture-guide (Accessed: 29 November 2021).

4. Jansons J et al. Vaccines (Basel). 2020 Jun 18;8(2):318. https://doi.org/10.3390/vaccines8020318

5. Glukhov A et al. J Clin Med. 2021 Mar 4;10(5):1055. https://doi.org/10.3390/jcm10051055.

### P5. Obtaining of influenza A virus mRNA-vaccines encoding artificial antigens using modified analogs of nucleotides

#### Sergey V. Sharabrin*, Andrey P. Rudometov A.P., Maria B. Borgoyakova, Victoria R. Litvinova, Larisa I. Karpenko

##### State Research Center of Virology and Biotechnology “Vector”, Koltsovo, 630559 Novosibirsk, Russia

###### Presenting author: Sergey V. Sharabrin (sharabrin_sv@vector.nsc.ru)

*Infectious Agents and Cancer* 2022, **17(Suppl 1)**: P5

**Background: **Nucleic acids-based influenza vaccines are promising platform which has been developed rapidly in recent times. We have previously constructed and studied the immunogenicity of DNA vaccines encoding artificial immunogens AgH1, AgH3, and AgM2, which contained conserved fragments of the hemagglutinin stem of two subtypes of influenza A—H1N1 and H3N2—and conserved protein M2. We have previously shown that immunization of BALB/c mice with a combination of DNA vaccines encoding these antigens induced both humoral and cellular responses, as well as a moderated statistically significant cross-protective effect against two heterologous viruses: A/California/4/2009 (H1N1pdm09) and A/Aichi/2/68 (H3N2) [1]. The aim of this study was to obtain and evaluate the immunogenicity of mRNA-vaccines encoding artificial influenza A virus antigens.

**Materials and methods: **For the synthesis of mRNA vaccine constructs, DNA plasmids encoding artificial antigens AgH1, AgH3, and AgM2 were used as templates. The mRNAs were synthesized using a highly effective in vitro RNA synthesis kit T7 mScript™ Standard mRNA Production System (CellScript, USA) with NTP mixes containing different NTP (A,G,C and U or Ψ). Polyadenylation and capping were carried out using a ScriptCap™ Cap 1 Capping System (CellScript, USA) and an A-Plus™ Poly(A) Polymerase Tailing Kit (CellScript, USA) according to the manufacturer’s recommendations. mRNA purification was carried out using a Monarch® Total RNA Miniprep Kit (New England Biolabs, USA). After synthesis, RNAs were mixed in equal concentration and the mixture of mRNAs was obtained containing (mRNA-AgH1 + mRNA-AgH3 + mRNAAgM2) (ΣmRNA). To evaluate ΣmRNA-vaccine immunogenicity we used BALB/c mice. Mice were divided into 3 groups with 6 animals in each group: group 1 (ΣmRNA with uridine), group 2 (ΣmRNA with Ψ), group 3 (negative control, normal saline). Mice were immunized with 45 μg RNA/100 μL (15 μg each immunogens) of normal saline intramuscularly in the upper thigh of the hind limb twice on days 0 and 21. On the 41st day, mice were bled for serum analysis by ELISA.

**Results and discussion: **The data showed that immunization of mice with pseudouridine-containing ΣmRNA induced statistically significant antibody titers against influenza A, while uridine-containing ΣmRNA generated low levels of antibodies. These data confirm the importance of mRNA modification with pseudouridine, which enhances the stability of the nucleic acid molecule and increases the level of mRNA translation. Our results showed that modified naked mRNA vaccines encoding artificial antigens constructed with conserved fragments of the hemagglutinin stem of influenza viruses, H1N1, H3N2, and the M2 protein, can induce a specific antibody response against influenza A virus in mice. In the next experiment, we plan to investigate the protective properties of the resulting mRNA vaccines.

**Acknowledgements: **The study was conducted under the state assignment of FBRI SRC VB “Vector”, Rospotrebnadzor.

**References**

1. Bazhan, S.; et al., Immunogenicity and protective efficacy of influenza a DNA vaccines encoding artificial antigens based on conservative hemagglutinin stem region and M2 protein in mice. Vaccines 2020, 8, 448.

### P6. Design of polyepitope HIV-1 immunogens including linear epitopes recognized by broadly neutralizing antibodies

#### Andrey P. Rudometov*, Nadezhda B. Rudometova, Viktoriia R.Litvinova, Lyubov A. Orlova, Alexander A. Ilyichev, Larisa I. Karpenko

##### State Research Center of Virology and Biotechnology “Vector”, Koltsovo, Novosibirsk Region 630559, Russia

###### Presenting author: Andrey P. Rudometov (rudometov_ap@vector.nsc.ru)

*Infectious Agents and Cancer* 2022, **17(Suppl 1)**: P6

**Background: **The HIV epidemic continues to be an acute problem of world health, and therefore research on the development of a preventive vaccine is relevant. A promising approach to development of a vaccine is the design of immunogens aimed at focusing the immune response on vulnerable regions of the virus, which are targeted, in particular, by broadly neutralizing antibodies (bnAbs). It is believed that highly conserved epitopes are present in those regions of viral proteins that are required for the "survival" of the virus. These regions can be considered as a promising epitopes for vaccine development. The aim of this work was to create artificial immunogens containing epitopes from conserved regions of HIV-1 that recognized by bnAbs.

**Results: **At the first stage, the theoretical design of 16 variants of polyepitope immunogens was carried out by combining the characterized B- and T-cell epitopes. 3 promising sequences were selected for synthesis and further study of the properties of polypeptides. The abbreviated names of these construct are 6.1.2, 9.2.5 and 10.1. The selected constructs include B-cell epitopes recognized by broadly neutralizing antibodies 4E10, 10E8, VRC34.01, a linear peptide that mimics the epitope recognized by the VRC01 antibody, and B-cell epitope from loop V3. The constructs include the following Th epitopes: gp160 (gp120, V2-loop, 167–182 a.a.), gp160 (gp120, 102–117 a.a.), gp160 (gp120, 421–436 a.a.), gp160 (gp41, 827–841 a.a.), gp160 (gp120, 443–459 a.a.) and gag (p24, 288–305 a.a.). Nucleotide sequences of promising variants were synthesized and cloned into the pET21 expression vector to produce recombinant proteins in a prokaryotic expression system. Bacterial producers of recombinant immunogens have been obtained. It was shown that protein 6.1.2 was found in the soluble fraction of E. coli cells, and two proteins (9.2.5 and 10.1) were found in inclusion bodies. Proteins 6.1.2 and 9.2.5 were purified and their secondary structures were characterized by circular dichroism spectroscopy. Immunochemical analysis showed that immunogens 6.1.2 and 9.2.5–9 containing the linear epitope 10E8 are recognized by the abovementioned monoclonal antibody bNAbs 10E8.

**Discussion and conclusions: **At present, mRNA technology is an intensively developing area in the field of vaccine development; therefore, we tried to obtain immunogen 6.1.2 also as a mRNA vaccine. The nucleotide sequence of 6.1.2 variant was optimized for expression in eukaryotic cells, synthesized and cloned into the plasmid vector pVAX. This plasmid was used as template for the synthesis of mRNA vaccine, coding 6.1.2 gene. Further studies will be aimed at investigating the immunogenicity of the resulting polyepitope constructs and their ability to induce the HIV-neutralizing antibodies in laboratory animals.

**Acknowledgements: **The reported study was funded by the Russian Foundation for Basic Research (project No. 20-04-00879) and the state assignment of FBRI SRC VB “Vector”, Rospotrebnadzor.

### P7. Tools and technologies for inducible and reversible packaging of CRISPR/Cas9 ribonucleoprotein complexes and their targeted delivery by biological nanoparticles

#### Sergey Brezgin^1,2^, Anastasiya Kostyusheva^1^, Natalia Ponomareva^1^, Ekaterina Bayurova^3,4^, Irina Goptar^5^, Anastasiya Nikiforova^5^, Anna Sudina^6^, Ilya Gordeychuk^3,4^, Olga Slatinskaya^7^, Georgy Maximov^7,8^, Anastasiya Frolova^9^, Vladimir Chulanov^1,2,9^ and Dmitry Kostyushev^1,2*^

##### ^1^National Medical Research Center of Tuberculosis and Infectious Diseases, Ministry of Health, 127994, Moscow, Russia; ^2^Scientific Center for Genetics and Life Sciences, Division of Biotechnology, Sirius University of Science and Technology, 354340 Sochi, Russia; ^3^Chumakov Federal Scientific Center for Research and Development of Immune-and-Biological Products of Russian Academy of Sciences, 108819 Moscow, Russia; ^4^National Research Center for Epidemiology and Microbiology named after NF Gamaleya, 123098 Moscow, Russia; ^5^Izmerov Research Institute of Occupational Health, Moscow, 105275, Russia; ^6^Federal State Budgetary Institution Centre for Strategic Planning and Management of Biomedical Health Risks of the Federal Medical Biological Agency, 119435, Moscow, Russia; ^7^Biophysics Department, Biological Faculty, Lomonosov Moscow State University, Leninskie Gory, 1/12, 119234 Moscow, Russia; ^8^Federal State Autonomous Educational Institution of Higher Education National Research Technological University “MISiS”, 119049 Moscow, Russia; ^9^Sechenov University, 119991 Moscow, Russia

###### Presenting author: Dmitry Kostyushev (dkostushev@gmail.com)

*Infectious Agents and Cancer* 2022, **17(Suppl 1)**: P7

**Background: **CRISPR/Cas9 systems enable precise manipulations with the genome which can be used for developing novel therapeutic approaches to treat hereditary disorders, cancer, combat viral infections etc. Complexes of Cas9 protein and single-guide RNA (sgRNA) (ribonucleoprotein complexes—RNPs) represent the most potent and specific approach for the use of gene editing technologies. However, delivering CRISPR/Cas9 RNPs in vivo is the major challenge and currently represents the biggest obstacle for moving CRISPR/Cas9 technologies into clinical trials. The aim of this study was to develop a new method for inducible and reversible packaging of CRISPR/Cas9 RNPs and test its antiviral activity at hepatitis B virus (HBV) in vitro models.

**Materials and methods: **Streptococcus thermophilus StCas9 protein (CRISPR locus 1) was genetically fused with a light-inducible dimerization domain and RNA hairpin-interaction domain. A second partner of light-inducible dimerization system was fused with an intraluminal part of a constitutive protein of biological nanoparticles. An array of HBV-targeting sgRNA with RNA hairpins was generated using genetic engineering. HEK293T cells were transfected with genetically modified StCas9-encoding plasmid. Functioning of StCas9 protein with an array of sgRNAs was analyzed by in vitro cleavage assay. Nanoparticles were produced from HEK293T cells, isolated and purified by anion exchange chromatography. Interaction of the dimerization domains upon light illumination and delivery of Cas9 protein were analyzed by confocal microscopy. Nanoparticles were characterized by DLS and zeta-potential measurement. Packaging of StCas9-mCherry protein into biological nanoparticles was analyzed by FACS after conjugation with nanoparticle-specific microspheres. Packaging of sgRNAs was measured by PCR. Antiviral activity of generated nanoparticles was estimated by treating HepG2 cells transfected with recombinant HBV genome (rcccDNA) with StCas9/sgRNA-loaded nanoparticles for 5 days. Functionalization of biological nanoparticles was performed by a genetically encoding truncated variant of CD63 protein with a liver-enriched peptide. In vitro uptake of nanoparticles was assayed by FACS in HepG2 cell line.

**Results: **Developed light-inducible dimerization system enabled tunable packaging of Cas9 protein and sgRNA. CRISPR/Cas9-loaded nanoparticles significantly reduced HBV replication intermediates in vitro. Functionalization of CRISPR/Cas9-loaded nanoparticles resulted in a sevenfold increase in targeting hepatoma cell lines.

**Conclusions: **We developed a system for simultaneous, inducible and reversible packaging of CRISPR/Cas9 RNPs and demonstrated its potent antiviral activity at HBV in vitro model. Functionalization of generated nanoparticles enables tissue-specific delivery of CRISPR/Cas9 RNPs.

**Acknowledgements: **This work was funded by RSF grant 20-15-00373.

### P8. Efficacy of co-transfection and levels of co-expression of two fluorescent reporter proteins in co-transfected cells

#### Elena Royo Rubio^1,2^, Ingrida Mitre^2^, Juris Jansons^3 *^.

##### ^1^Gregorio Marañón Health Research Institute, Madrid, Spain; ^2^Riga Stradins University, Institute of Oncology, Riga, Latvia; ^3^ Latvian Biomedical Research and Study Centre, Riga, Latvia

###### Presenting author: Juris Jansons (Jansons@biomed.lu.lv)

*Infectious Agents and Cancer* 2022, **17(Suppl 1)**: P8

**Background: **The in virto tests based on introducing into the cells the plasmids expressing reporter and test proteins, for example evaluating the ability of the proteins to induce cytokines synthesis by measuring expression of a reporter, commonly luciferase, driven by a specific cytokine promoter, are popular because of their simplicity, fastness, and possibility to run the assay in the high throughput screening format. However, the reliability and reproducibility of the results of such tests are very dependent on the efficacy of plasmid cotransfection, as well as proportion of transfected cells receiving both plasmids. The aim of this study was to evaluate the efficacy of plasmid delivery into eukaryotic cells by transfection and to find the ratio between two plasmids optimal for efficient co-delivery into the same cells, as well as for efficient expression of the reporter.

**Materials and methods: **Study used plasmids pGFP expressing Green Fluorescent Protein (GFP) and piRFP670 expressing near-infrared iRFP670 fluorescent reporter protein under control of IECMV promoter [1] (kind gift of prof. Vladislav Verkhusha, USA). HEK293T cells were seeded onto 24 well plates (3 × 10^5^ cells per well) 24 h pre-transfection. The commercial polyalkyleneimine cation transfection reagent Turbofect™ (Thermo Fisher Scientific) was used according to manufacturer recommendations. Cells were collected 48 h post-transfection. Cells expressing reporters were detected by flow cytometry using the FACSAriaII (Becton Dickinson) analyzer to data acquisition and FlowJo v10 software to the data analysis.

**Results: **We simulated in vitro the scenario of using “test” and “reporter” plasmids, using one as a test, and the other as the actual reporter protein. As the reporter we have chosen plasmid expressing GFP (pVaxGFP) and as a test protein, iRFP670 protein (pVaxiRFP670). Transfection mixes were formulated using two doses of pVaxGFP-0.8 and 0.4 μg, alone or mixed with pVaxiRFP670 taken in amount 0.1, 0.2, 0.3 and 0.4 µg. The proportion of GFP positive cells-GFP-expressing HEK cells was the same (86–93%) for both 0.8 and 0.4 µg of the GFP plasmid taken for the transfection alone. Further, the proportion of co-transfected/co-expressing cells was fluctuating in the range of 75–83% independently on the amounts of GFP and iRFP670 encoding plasmids used in the transfection mixtures. Analyzing relative fluorescence of the double GFP and iRFP670 positive cells, we found that an increase in the amount of pVaxiRFP670 in the mixture (from 0.1 µg to the 0.4 µg) led to respective increase in the expression of iRFP670 up to two-fold. At the same time, the level of expression of GFP in the double GFP and iRFP670 positive cells dramatically decreased (up to 90%) comparing to the level of GFP expression in HEK cells transfected with pVaxGFP alone. Decrease did not depend on the amount of iRFP670 plasmids used in co-transfection.

**Discussion and conclusions: **Turbofect™ transfection reagent ensures efficient co-delivery of plasmid mixtures into one and the same cells at diverse ratios of plasmids in the mixture (up to 1:8). This demonstrates that the level of reporter expression can be reliably used to assess activity of test plasmid in a eukaryotic cell after co-transfection. Experiments demonstrated toxicity of iRFP670 to expressing cells, at least on the level of interference with expression of other reporters. It may relate to extreme stability of iRFP670 [2] which may result in induction of ER stress, namely unfolded protein response which halts protein synthesis [3]. Our results demonstrate that it is necessary to keep in mind that test protein can negatively affect the metabolism of the target cell causing an unspecific loss of the reporter signal, unrelated to the activity to be measured by reporter assay, resulting in incorrect interpretation of test results.

**Acknowledgments: **Latvian FLPP project “Pathogenicity of human Hepatitis C virus relying on enzymatic properties of viral RNA-dependent RNA-polymerase” (agreement nr lzp-2020/2-0376), Program NAWA EUROPARTNER: Strengthening and spreading international partnership activities of the Faculty of Biology and Environmental Protection for interdisciplinary research and innovation of the University of Lodz.

**References**

1. Shcherbakova DM, Verkhusha VV. Near-infrared fluorescent proteins for multicolor in vivo imaging. Nat Methods. 2013 Aug;10(8):751–4. https://doi.org/10.1038/nmeth.2521. Epub 2013 Jun 16.

2. Matlashov ME, Shcherbakova DM, Alvelid J, Baloban M, Pennacchietti F, Shemetov AA, Testa I, Verkhusha VV. A set of monomeric near-infrared fluorescent proteins for multicolor imaging across scales. Nat Commun. 2020 Jan 13;11(1):239. https://doi.org/10.1038/s41467-019-13897-6.

3. Hetz C, Papa FR. The Unfolded Protein Response and Cell Fate Control. Mol Cell. 2018 Jan 18;69(2):169–181. https://doi.org/10.1016/j.molcel.2017.06.017. Epub 2017 Nov 5.

### P9. Development of questionnaire for assessment of higher nervous activity: pilot study in pediatric post cancer

#### Vladimir N. Kasatkin^1*^, Alena A. Deviaterikova^1,2^, Alexander F. Karelin^2^

##### ^1^Peoples’ Friendship University of Russia (RUDN University); ^2^Research Clinical Rehabilitation Center «Russkoe Pole» Federal Research Clinical Center of Pediatric Hematology, Oncology and Immunology named after D. Rogachev, Moscow

###### Presenting author: Vladimir N. Kasatkin (ryabovaalenaandreevna@gmail.com)

*Infectious Agents and Cancer* 2022, **17(Suppl 1)**: P9

**Background: **Treatment of viral infections and cancer often leaves behind late effects. Late effects can include disruption of the higher nervous activity, body pain, headache, and other symptoms that reduce the quality of life of children [1]. Somatic complaints are often not evaluated by doctors unless they are symptoms of cancer recurrence [2]. In clinical practice, doctors use various scales and questionnaires to assess the presence of symptoms of disruption of the higher nervous activity, body pain, headache and their severity [3]. However, these instruments are used mainly among adults; such instruments for pediatric cancer and other infectious diseases affecting the nervous system are missing [4]. The aim of this study was to create a questionnaire that assesses the presence and severity of somatic symptoms, as well as their impact on the child's daily activity.

**Material and methods: **Patient cohort: 515 children (243 girls and 272 boys) survivors of oncological diseases of the central nervous system.

Research procedure: We created the technique in several stages. At the first stage, we selected a pool of statements—the "zero" version of the questionnaire. This version of the questionnaire was then evaluated by 11 expert psychologists for a qualitative assessment of the statements. In the course of the peer review, some of the statements were deleted, and some were changed. The resulting first version of the questionnaire carried out on 25 patients. We then conducted a massive survey among patients who agreed to take part in the study.

Statistical analysis: To assess the reliability of the questionnaire, we used the Cronbach alpha test of internal consistency. To assess the normal distribution, we used visual tests and the Shapiro–Wilk test. To assess the criterion validity of the questionnaire we used Spearman’s correlation with the Thomas Achenbach questionnaire. To process the results, we used SPSS ver. 22.

**Results: **The final questionnaire includes 28 items. The measure of the adequacy of the Kaiser-Mayer-Olkin (KMO) sample was 0.870.

Reliability: the overall Cronbach alpha for the questionnaire scales was 0.816. Cronbach's alpha for the first factor was 0.811; for the second factor − 0.795; for the third factor − 0.772; for the fourth factor − 0.673; fifth factor − 0.674; sixth factor − 0.629.

Construct validity: we found significant Spearman correlations between the questionnaire scale and the Thomas Achenbach questionnaire. Thus, a comparison of the two questionnaires showed the following connections: the “symptoms” scale from CPCT is associated with the “somatic problems” scale from the Thomas Achenbach questionnaire (r = 0.421); the scale of "negative emotions" is associated with the scale of "anxiety" (r = 0.400); the decreased activity scale is related to the overall score.

**Discussion: **Many viruses infect brain and may cause meningitis, encephalitis, meningoencephalomyelitis, Guillian-Barré-like-syndromes as well as strokes [5]. Human immune deficiency virus, flaviviruses, SARS-CoV-2, influenza, and human herpes viruses cause low-grade neuroinflammation which interferes with physiological repair and remodeling processes in the brain and induce and/or accelerates brain aging and causes cognitive dysfunctions [6, 7]. Questionnaires as CPCT would be helpful in the assessment of somatic state, higher nervous activity and cognitive functions in children suffering from chronic viral infections.

**Conclusion: **We have developed a multifactorial symptom assessment questionnaire ("Children's post-cancer test" -CPCT). It helps to assess the presence of negative symptoms, their severity and impact on the daily activity of communication and emotions in children who survivor’s cancer, and may be used to assess higher nervous activity in children suffering from chronic viral infections.

**References**

1. Zoëga S. et al. Pain and other symptoms and their relationship to quality of life in cancer patients on opioids. Quality of Life Research 2013; 22(6),1273–1280.

2. Stone A.L. et al. Topical review: Pain in survivors of pediatric cancer: Applying a prevention framework. Journal of Pediatric Psychology 2018; 43(3), 237–242

3. "Neurological Diagnostic Tests and Procedures", NINDS. April 10, 2019. NIH Publication No. 19-NS-5380. Available at https://www.ninds.nih.gov/Disorders/Patient-Caregiver-Education/Fact-Sheets/Neurological-Diagnostic-Tests-and-Procedures-Fact (Accessed: 29 November 2021).

4. Shi Q. et al. Using a symptom-specific instrument to measure patient-reported daily functioning in patients with cancer. European Journal of Cancer 2016; 67, 83– 90.

5. Meyding-Lamadé U et al. Emerging and re-emerging viruses affecting the nervous system. Neurol Res Pract. 2019 Jun 11;1:20. https://doi.org/10.1186/s42466-019-0020-6.

6. Filgueira L et al. The Influence of Virus Infection on Microglia and Accelerated Brain Aging. Cells. 2021; 10(7):1836. https://doi.org/10.3390/cells10071836.

7. Sun B. et al. Differential cognitive impairment in HCV coinfected men with controlled HIV compared to HCV monoinfection. J Acquir Immune Defic Syndr. 2013; 62(2), 190-6. https://doi.org/10.1097/QAI.0b013e31827b61f1.

